# Evaluating Nurses' Perspectives on the Acceptability and Practicality of Comfort Rounding for Personalised Nutritional and Mobility Care in Surgical Wards: A Mixed‐Methods Feasibility Study

**DOI:** 10.1111/jan.70462

**Published:** 2025-12-21

**Authors:** Femke Becking‐Verhaar, Fanny Pelzer, Harm Van Noort, Cariline Roosen, Yvonne Van Eijk‐Hustings, Marian De Van Der Schueren, Hester Vermeulen, Getty Huisman‐De Waal

**Affiliations:** ^1^ Department of Surgery Radboud University Medical Centre Nijmegen the Netherlands; ^2^ Department of Nursing Maastricht University Medical Centre Maastricht the Netherlands; ^3^ Department of Clinical Epidemiology and Medical Technology Assessment (KEMTA) Maastricht University Medical Centre Maastricht the Netherlands; ^4^ HAN University of Applied Sciences Nijmegen the Netherlands; ^5^ Wageningen University and Research Wageningen the Netherlands; ^6^ IQ Health Radboud University Medical Centre Nijmegen the Netherlands

**Keywords:** comfort rounding, fundamental care, hourly rounding, intentional rounding, mobilisation, mobility care, nutrition, nutritional care, patient participation, qualitative research

## Abstract

**Aim:**

To evaluate nurses' perspectives on factors influencing the acceptability and practicality of comfort rounding, focussing on personalised nutritional and mobility care.

**Design:**

Mixed‐methods feasibility study.

**Methods:**

Focus group interviews with nurses were conducted before, during and at the end of the implementation period (2022–2023). A questionnaire assessed acceptability and practicality among nurses at the end of the implementation. Data were analysed using directed content analyses and descriptive statistics.

**Results:**

Comfort rounding's acceptability and practicality were influenced by nurses' attitudes, knowledge and skills, patient characteristics and the nurse–patient relationship. Barriers included workload, time pressure, team culture and the extensive, rigid design of comfort rounding. Questionnaire responses demonstrated nurses perceived added value of comfort rounding and frequently engaged patients in activities related to nutrition and mobility. However, it was not performed as originally intended.

**Conclusion:**

Nurses considered personalised nutritional and mobility care important and frequently provided it during ‘usual care’. However, nurses were critical of comfort rounding's acceptability and practicality and did not perform it as intended.

**Implications for the Profession and/or Patient Care:**

Comfort rounding's concept does not align well with current nursing practice. Greater tailoring to nurses' preferences or alternative approaches to structuring personalised nutritional and mobility care are recommended.

**Impact:**

*What problem did the study address:* Hospitalised patients often receive suboptimal nutritional care and are largely inactive. The challenge is to integrate personalised nutritional and mobility care effectively into standard nursing practice to enhance patient safety and well‐being. Comfort rounding could improve patient safety and satisfaction; however, there is no research evaluating the feasibility of comfort rounding in relation to personalised nutritional and mobility care. *What were the main findings:* Comfort rounding was generally perceived as valuable and aligned with existing care routines, but its rigid structure was often considered impractical. Comfort rounding was not performed as originally intended due to the influence of individual, social and organisational factors. Flexibility in execution emerged as a critical factor for successful integration. *Where and on whom will the research have an impact?:* Comfort rounding can enhance attention to nutrition, mobility and patient participation when adapted to local contexts and delivered with flexibility. Policymakers and nurse leaders should avoid rigid protocols and instead support tailored implementation strategies alongside the practical delivery of locally tailored interventions.

**Reporting Method:**

Consolidated criteria for reporting qualitative research and Checklist for Reporting of Survey studies.

**Patient or Public Contribution:**

Nurses were involved in all stages of the study, contributing through focus group interviews and completing a questionnaire to help develop and evaluate comfort rounding.

**Trial Registration:**

PaNaMa Research Management System, number 112832

## Summary

- What does this paper contribute to the wider global clinical community?
  ○ Comfort rounding may support fundamental care delivery.
  ○ Acceptability and practicality of comfort rounding depend on flexibility.

## 1 Introduction

According to the Fundamentals of Care framework (FoC), nurses are in a key position to strive for safe, effective and high‐quality care within the physical, psychosocial and relational domain incorporating nutritional and mobility care and patient participation (Kitson 2018; Mitchell 2008). Several studies demonstrated that attention for early nutrition and mobilisation, as well patient participation, could reduce the risk of post‐surgical complications and positively affect patients' recovery (Koenders et al. 2021; Koenders et al. 2020; Ljungqvist and Hubner 2018; van den Berg et al. 2021). Furthermore, it could enhance patients' psychological and social well‐being (Kalisch et al. 2014; Koenders et al. 2021; Rios et al. 2021). However, nutritional and mobility care are vulnerable to care left undone (van Belle et al. 2020; Van Den Berg et al. 2023), hospitalised patients sometimes receive suboptimal nutritional care (Ten Cate et al. 2021) and hospitalised patients remain largely inactive (Fazio et al. 2020). So, the challenge is to integrate nutritional and mobility care and patient participation more effectively into standard nursing practice to enhance patient safety and well‐being. This could be achieved through comfort rounding (CR)—predefined and structured rounds where nurses visit their patients with a focus on nutrition, mobility and patient participation on predetermined time intervals and with a standardised protocol during their shifts (Harris et al. 2019; Sims et al. 2018). This study evaluates the acceptability and practicality of CR from nurses' perspectives.

## 2 Background

Recovery from major abdominal surgery is challenging due to complications which occur in 30%–40% of the patients. Complications are the main cause of high morbidity and mortality, prolonged hospital stays and reduced life expectancy (Ferreyra et al. 2009; Jakobson et al. 2014; Khuri et al. 2005; Veličković et al. 2020).

Studies have demonstrated that approximately 10% of surgical patients are malnourished (Kruizenga et al. 2016; Ten Cate et al. 2021) and hospitalised patients often receive suboptimal nutritional care due to competing priorities and ward regulations (Ten Cate et al. 2021). Furthermore, evidence indicates that hospitalised patients remain largely inactive, with sedentary behaviour accounting for 87%–100% of their day (Fazio et al. 2020). These factors adversely affect recovery after surgery. The Enhanced recovery after surgery (ERAS) guidelines aim to improve recovery by reducing perioperative stress responses and enhancing postoperative outcomes. Within the multidisciplinary team—including surgeons, anaesthesiologists and nurses—nurses play a pivotal role throughout the preoperative and postoperative phases (Ljungqvist and Hubner 2018; Visioni et al. 2018). In particular, during the postoperative phase, nurses are essential in reducing complications and promoting recovery by prioritising early nutrition and mobilisation, both key components of the ERAS approach (Ljungqvist and Hubner 2018). Early nutrition and mobilisation contribute significantly to enhanced recovery: lowering the incidence of physical complications and mortality following surgery (Correia and Waitzberg 2003; EPUAP et al. 2019; Nydahl et al. 2023; van der Leeden et al. 2016), while also positively impacting patients' psychological and social well‐being (Kalisch et al. 2014; Koenders et al. 2021; Rios et al. 2021) and functional capacity postoperatively (de Almeida et al. 2017). Collectively, these findings underscore the indispensable role of nurses in delivering effective nutritional and mobilisation care to support surgical recovery.

According to the Fundamentals of Care framework (FoC), nurses are in a key position to strive for safe, effective and high‐quality care within the physical, psychosocial and relational domain (Kitson 2018; Mitchell 2008). Attention for early nutrition and mobilisation is encompassed in the physical domain of the FoC framework (Feo et al. 2018; Feo et al. 2017; Kitson 2018) and could be supported by activities in the psychosocial and relational domain, incorporating patient participation. Patient participation is defined as an interactional process between nurses and patients, in which mutual negotiation and patients gaining insight through consideration are the core elements (Larsson et al. 2007; Sahlsten et al. 2007). Patient participation can enhance nutritional care by identifying patients' eating difficulties and dietary preferences (van den Berg et al. 2021) and individual patient education and goal setting could lead to improved physical performance and body weight of patients (Van Den Berg, Vermeulen, et al. 2023). Furthermore, patient participation can improve mobility care by discussing, setting and achieving physical activity goals through tailored interventions that consider patients' mobility needs and preferences (Koenders et al. 2021; Koenders et al. 2020). Despite the aforementioned importance of nurses actively approaching and involving patients across all domains of FoC, nurses still tend to focus on task‐oriented activities primarily within the physical domain, which reflects nurses' biomedical focus. Furthermore, it is not always evident that nurses consistently incorporate patients' needs and experiences, engage in discussions about care planning, or actively facilitate patient participation in these processes (van Belle et al. 2020; van den Berg et al. 2023). Moreover, patient mobilisation and certain aspects of nutritional care are often at risk of being left undone (Griffiths et al. 2018). Patients themselves also do not always take the initiative, due to a lack of awareness about the risks of malnutrition or uncertainty regarding mobilisation, which can lead to physical inactivity and highlights the need for professional support (Koenders et al. 2020; Van Den Berg, Vermeulen, et al. 2023). Therefore, a key challenge is to systematically embed nutritional and mobility care and patient participation structurally into the organisation of daily nursing practice. To this end, CR has emerged as a possible solution, with specific attention to nutrition, mobility and patient participation. Studies have demonstrated that CR can enhance patient safety (e.g., fall prevention) and patient satisfaction (Daniels 2016; Di Massimo et al. 2022), as well as reduce nurses' workload and foster a more supportive and efficient work environment (Ryan et al. 2019).

## 3 The Study

### 3.1 Aim

In this study, CR with structured focus on nutrition, mobility and patient participation is developed, tailored and implemented within the organisation of nursing practice on two surgical wards in two university hospitals for a one‐year period. The implementation is guided by an implementation framework (Grol et al. 2013). This study aims to evaluate nurses' perspectives on the acceptability and practicality of CR, with specific attention to nutrition, mobility and patient participation in nursing practice.

## 4 Methods

### 4.1 Design

In this mixed‐methods feasibility study, the focus was on to evaluate whether CR can and does work in nursing practice and to discover barriers and facilitators. Therefore, two areas of focus were studied as primary outcomes: the ‘acceptability’ and ‘practicality’ of CR (Bowen et al. 2009). Evaluating acceptability would demonstrate nurses’ reactions to CR, its appropriateness and fit in daily nursing practice, nurses’ satisfaction with CR and their willingness to use CR. Evaluating practicality would reveal how easily CR could be executed by nurses and which facilitating and hindering factors are contributing to this (Bowen et al. 2009). Focus group interviews (FGs) were used to evaluate nurses’ experiences and were held at four moments (see Table 1). This design allowed for data comparison across baseline and follow‐up measurements and to determine if slight differences appeared over time. After FG T3, an expert‐based questionnaire was distributed to all nurses. This questionnaire was used as a supplementary tool alongside the FGs and aimed to evaluate the acceptability and practicality of CR among a larger group of nurses. Therefore, the results of the questionnaire were used to complement the findings from the FGs. Consolidated criteria for reporting qualitative research (COREQ) were used for reporting the qualitative part of the study (Tong et al. 2007) (see Appendix E) and the Checklist for Reporting of Survey studies was used to report the questionnaire study (Sharma et al. 2021) (see Appendix F).

**TABLE 1:** Timeline of data collection and activities regarding the study.

Study stages^b^	T0	Implementation	T1	T2	T3
Period	September 2021–February 2022	March 2022	April–May 2022	June–December 2022	January–May 2023
Measurement	Baseline measurement		Intermediate measurement	Intermediate measurement	Follow‐up measurement
Data collection	Focus group interview T0 (February 2022)		Focus group interview T1 (April 2022)	Focus group interview T2 (December2022)	Focus group interview T3 (April 2023) Questionnaire (May 2023)
Implementation phase^a^	Orientation, Insight	Insight, Acceptance, Change	Change	Change	Sustainability
Comfort rounding (CR)	Preparing and constructing CR	Tailoring CR, Introduction of CR	Tailoring CR	Tailoring CR	Evaluation of CR
Activities	Informing colleagues in different ways, collecting nurses' opinions about CR	Giving feedback from the focus group interview T0 to nurses, discussing results, constructing CR with input and feedback of colleagues	Data collection to collect experiences, tailoring CR.	Data collection to collect experiences, tailoring CR	Data collection to evaluate CR

^a^ References: Grol et al. (2013).

^b^ The study comprised four stages: T0 (baseline measurement), T1 and T2 (intermediate measurements) and T3 (follow‐up measurement).

### 4.2 Study Setting

The study spanned from September 2021 to September 2023, with CR implemented for 1 year on two surgical wards in two university hospitals in The Netherlands. The first ward comprised 40 beds, 35 qualified and registered nurses, either vocationally or bachelor‐educated (numbers of each not registered), 3 clinical team leaders and patients were mainly admitted for abdominal surgery with underlying gastroenterological, oncological, or gynaecological diseases (e.g., colorectal surgery, debulking and uterus extirpation). Nurse–patient ratio in the day shift was 1:8 and in the evening shift 1:12. The used electronic patient file was Oracle Cerner (www.cerner.com). The ward of this hospital is referred to as ‘Hospital 1’. The second ward, referred to as ‘Hospital 2’ comprised 18 beds, 33 qualified and registered nurses—11 vocationally educated and 22 holding a bachelor's degree—2 clinical team leaders and patients were mainly admitted for elective major abdominal‐oncological surgery (e.g., Hyperthermic Intraperitoneal Chemotherapy, pancreaticoduodenectomy, esophagectomy, hemi hepatectomy, abdominal perineal resection). Nurse–patient ratio in the day shift was 1:3 and in the evening shift 1:6. The used electronic patient file was EPIC (www.epic.com). In both hospitals, there were no differences in care activities or responsibilities between vocationally‐educated nurses and those with a bachelor's degree during the study period. Furthermore, both educational levels shared the same statutory registration in the national register for healthcare professionals at that time. Thus, all nurses worked within a single team without clear demarcation of responsibilities.

Both hospitals were equipped with allied health professionals such as physiotherapists and dieticians. Although both centres are university hospitals, there are differences in the nurse–patient ratio. Multiple factors may be involved. One possible explanation for the variation in nurse–patient ratio is the difference in surgical procedures and complexity. The participating department at Hospital 1 performs relatively more colorectal surgeries, which are often considered less complex than the extensive pancreatic and liver surgeries more commonly performed at Hospital 2. Additionally, the determination of staffing ratios was also influenced by the internal organisational structures of the individual hospitals.

### 4.3 Sampling

A number of five to eight participants was targeted for each focus group interview. Nurses of both hospitals were invited by e‐mail to participate in the FGs when they met inclusion criteria. Reminders were sent after 1 week each time. A convenience sampling approach was used: all nurses who voluntarily chose to participate were able to do so, in order to obtain a broad range of experiences within the heterogeneous groups of nurses. Nurses willing to participate provided informed consent before the FG. At T3, all nurses from both hospital wards who met inclusion criteria were invited to voluntarily complete a one‐time anonymous questionnaire. A convenience sampling method was used for the questionnaire as well, striving for as many as possible respondents.

### 4.4 Inclusion Criteria

For the FGs and questionnaire, similar criteria for nurses applied:

– Graduate nurses working on a regular basis on the specific ward
– Nurses with ≥ 3 months' work‐experience on the specific ward
– Nurses working in direct patient‐related care

### 4.5 Study Intervention: Development and Implementation of Comfort Rounding

CR refers to structured visits (rounds) nurses make to patients at set times, following a standardised protocol (Sims et al. 2018). In this study, CR was developed and implemented at both surgical wards and meant that nurses would speak regularly with their patients about mobilisation and nutrition (e.g., the importance of both to recover from surgery, support needed) and how patients wanted to participate. This process was structured by the implementation strategy of Grol et al. (2013) and included the phases orientation, insight, acceptance, change and sustainability. These phases guided the implementation activities as described in the row ‘Activities’ in Table 1. Although this implementation strategy was used to guide the introduction of CR on both wards, this study was explicitly not classified as implementation research. Before proceeding with full implementation, the aim of this study was to first explore the acceptability and practicality of CR.

At study start, each hospital developed the content and structure of CR for both day and evening shifts, based on feedback from FG T0 (see Appendix A). The structure of CR consisted of time blocks during which nurses were expected to talk with patients about certain topics or take specific actions related to mobility or nutrition. CR was continuously tailored in both settings to fit the needs and possibilities of the particular context and tailoring was guided by findings from FG T1 and T2 (see Table 2). In comparison, usual care in both hospitals involved regular rounds during which vital signs were measured, fluid balances were recorded and medication was administered, but structured attention (i.e., at predetermined time intervals and following a standardised protocol) to nutritional intake, mobility and patient participation related to these aspects, as defined to the definition of patient participation, was not present yet.

**TABLE 2:** Development and tailoring of comfort rounding (CR).

Study stage^a^	Hospital 1	Hospital 2
**After T0**	A schedule and content for CR was drafted at the request of nurses The schedule was visibly placed (e.g., computers, nurses' stations)	A schedule and content for CR was drafted in co‐creation with nurse colleagues The schedule was visibly placed (e.g., computers, nurses' stations)
**After T1**	Patients received the same schedule as nurses at the wall of their room Nurses received a teaching moment Information about CR was placed on digital screens in the hallway	A smart phrase was introduced in the nurses' reports of the patient file to remind nurses to report on their patients' nutritional intake and mobility Nurses no longer needed to adhere to the fixed schedule as they indicated that they needed more freedom to plan attention to nutrition and mobility themselves Reminders to perform CR were arranged (e.g., quiz, newsletter)
**After T2**	Nurses received reminders to perform CR (e.g., discussion moment, emphasis on CR during day start and day end, tailored information on screens in hallways)	The dietician provided teaching moments on nutrition on nurses' request The smart phrase in nurses' reports was changed because nurses indicated that the ‘old’ smart phrase resulted in double reporting about nutrition and mobility Some nurses indicated they wanted an extra reminder to perform CR in the patient file, some did not. To investigate what the majority wanted, a poll was conducted through the mail, which resulted in the rejection of an additional reminder in the patient file

^a^ T0 is the ‘baseline measurement’, T1 and T2 are ‘intermediate measurements’.

### 4.6 Data Collection

FGs offered the possibility to collect a range of nurses' perceptions and points of view about CR in a limited period through group interactions and discussions (Krueger 2014; Morgan 1996). Therefore, eight FGs with nurses were held (see Table 1). FG results reflected the views of a limited subset of the total number of nurses. To gather comprehensive feedback on nurses' CR performance and the acceptability and practicality of CR among a larger group of nurses, an expert‐based questionnaire was distributed to all nurses of both wards after FG T3 (see Table 1). Since it was clear beforehand that there were no differences in care activities or responsibilities between vocationally‐educated nurses and nurses with a bachelor's degree during the study period, it was decided not to distinguish between educational levels when composing the participants for the focus group interviews, distributing the questionnaires, or analysing the data.

#### 4.6.1 Focus Group Interviews

An interview guide and two semi‐structured topic lists, one for the baseline (T0) and one for the intermediate and follow‐up measurements (T1–T3), were constructed by female researchers FB, FP, GH and YvE. Both lists were based on four factors influencing the implementation of an innovation: individual, social, organisational and societal factors (Wensing et al. 2013a) (Appendix B). The topics encompassed how nurses performed nutritional and mobility care, how they involved patients and specific questions about CR, which varied between the topic lists of T0 and T1–T3. At T0, nurses were asked about their perceptions and assessments of the forthcoming CR implementation. At T1–T3, discussions focused, among other things, on the introduction and execution of CR by nurses. FGs in both hospitals were conducted in quiet locations in the hospital, audio‐recorded and transcribed verbatim. They were moderated by female early‐career researchers FP (nurse practitioner, MANP) and FB (nurse scientist, PhD candidate), who were trained in qualitative methods and by trained and supervised female nursing students. Only the researchers and participants were present. Field notes were sometimes, so not structurally taken. Data were pseudonymised and transcripts were shared with participants for member checking to ensure accurate representation of nurses' perspectives. The participants had no feedback on the transcripts.

#### 4.6.2 Questionnaire

A 27‐item questionnaire was made by six experts in the domain of nursing care (YvE, HvN, FP, GH, FB, AS) and one statistician (LB). See Appendix C.

The first six questions gathered respondent characteristics. Question seven asked if respondents were familiar with CR; if so, respondents were asked to answer questions 8–11. Question 8 and 11 consisted of seven, respectively, nine statements. Questions 9 and 10 asked how often CR were performed. Questions 9 to 11 were answered using a five‐point Likert scale, ranging from 1 (‘totally disagree’ or ‘never’) to 5 (‘totally agree’ or ‘always’). Questions 8.4 and 8.7 were negatively formulated; the answers to these questions were reverse‐coded because a higher score on each question indicated a more positive attitude to CR. If respondents were not familiar with CR, they were asked to respond to the open‐ended questions 12 and 13 to support the researchers with additional information. Questions 12 and 13 were excluded from this study as they did not contribute to assessing the acceptability and practicality of CR. The questionnaire was administered on paper for 2 weeks. Data were processed anonymously.

#### 4.6.3 Data Saturation

As this was a feasibility study designed to generate initial insights into the topic, the aim was to obtain in‐depth and meaningful data about nurses' experiences with CR. To achieve this, 1 FG was conducted per hospital at each measurement. Consequently, it is uncertain whether data saturation was reached. No power analysis was conducted for the questionnaires since the aim was to collect as many responses as possible through convenience sampling.

### 4.7 Data Analysis

#### 4.7.1 Analysis Focus Group Interviews

Data of the FGs were analysed with directed content analysis. A strength of this kind of qualitative content analysis is that data are coded, divided, analysed and identified systematically (Hsieh and Shannon 2005). Data were structured directly into fixed preliminary codes, differentiating between quotations that referred to a facilitating, hindering or neutral quotation and assigned to fixed referring themes: mobility, nutrition, comfort rounding specific, or general aspects. An open coding was then adjusted to provide more information about the quote. Subsequently, codes were assigned to factors that influenced the implementation of an innovation: individual, social, organisational and societal factors (Wensing et al. 2013b). These predetermined factors served as a framework to structure the results and offered the possibility to compare findings between the eight FGs and different studies (Assarroudi et al. 2018). Data were stored and analysed with the computer‐assisted qualitative data analysis software program ATLAS.ti (ATLAS.ti Scientific Software Development GmbH, Berlin, Germany, version 23.0.7.0) and Excel (Microsoft 365, version 2402).

#### 4.7.2 Analysis of the Questionnaire

Data of the questionnaire were processed in SPSS (IBM SPSS Statistics, version 29.0.0.0) and data were analysed with descriptive statistics.

### 4.8 Ethical Considerations

This study was cleared for approval by the ethics committees in both centres (Hospital 1 METC 2021‐2928, Hospital 2 METC 2021‐13334) because it was assessed as not subject to the Medical Research Involving Human Subjects Act. The study was carried out in accordance with the Declaration of Helsinki (World Medical Association 2018) and the Netherlands' General Data Protection Regulation. Following local data management rules, study data were securely stored physically or digitally for 10 years, accessible to four researchers (FP, YvE, GH and FB).

### 4.9 Rigour and Reflexivity

FGs were first coded by FB and FP (T0 and T2) and the bachelor's or master's students (T1 and T3). To increase the study's reliability, all focus group interviews were double coded independently from the first coding: FB and FP (T1 and T3), HvN and YvE (T0) and CR and YvE (T2). Initial coding and assignment to the four factors were discussed and consensus was reached among the researchers (CR, FB, FP, YvE) after which a consensus document was drafted.

## 5 Findings

In total, 32 instances of participation in focus group interviews were recorded, but some nurses participated more than once. Due to the use of convenience sampling, some overlap occurred and the total number of unique nurse participants was 26. The FGs lasted between 34 and 70 min, with 3–5 nurses participating in each interview. Most participating nurses were female (See Appendix D, Table 1). Results of FG T0 were described separate from FG T1–T3 as these results illustrated ‘usual care’ before the introduction of CR. FG T1–T3 demonstrated results over time and were described integrative and per factor. When results were specific to one of the hospitals or one of the FGs, this was made explicit in the text or citation. Otherwise, it was applicable to both hospitals or all FGs. Furthermore, experiences were acquired by a questionnaire completed by nurses of both wards at T3 (see Appendix D, Table 2). At Hospital 1, 21 nurses (60%) and at Hospital 2, 22 nurses (67%) responded. Most respondents (76%) of Hospital 1 and all respondents (100%) of Hospital 2 were aware of CR and answered therefore question 8 to 11 of the questionnaire. Results of the FGs and questionnaire were presented separately; the FGs first and the questionnaire thereafter.

### 5.1 Baseline Measurement: Focus Group Interviews T0

Before CR was introduced, nurses had positive expectations of structural attention to nutritional and mobility care because it could promote patient recovery and independence. CR was expected to be acceptable because nutritional and mobility care were already integrated into the day's structure. However, time investment was an expected barrier to perform CR according to nurses of Hospital 2. These nurses indicated that CR has to be performed on top of the other work and could be skipped:

I think we already do it partly, but we don't schedule specific moments for it. I think we are always somewhat involved with the patient's eating and drinking and organising it, but we don't really involve the patient in it throughout the day. I think we could gain a lot from that, but as for time planning, we'll have to see. (Respondent 2, Hospital 2, FG T0)

Individual characteristics of nurses (e.g., knowledge, experience, priority‐setting) influenced the structure, amount and manner in which nurses paid attention to their patients' nutritional and mobility care and this varied between nurses. Nurses' knowledge and skills were seen as supportive and important to facilitate personalised nutritional and mobility care and several patient characteristics (e.g., motivation) were mentioned as hindering or facilitating nutritional and mobility care.

### 5.2 Focus Group Interviews T1–T3: Individual Factor

#### 5.2.1 Nurses' Attitude

During the implementation period, nurses stated that they already paid attention to their patients' nutritional intake and mobility in daily routines by observing, guiding, informing, advising, setting goals, motivating and encouraging. CR did not appear to have any impact on these practices. However, CR may have influenced nurses at Hospital 2, as they furthermore mentioned discussing patients' needs, alternatives and nurses' expectations. Additionally, they made appointments with patients and involved relatives when necessary. Structure, amount and manner in which nurses paid attention to their patients' nutritional and mobility care still differed between individual nurses during the implementation period. These differences could be explained by individual characteristics of nurses such as attitude, age, skills, knowledge, experience, awareness and the time the nurse felt was available to work on it.

… the older nurses, yes, they have been in the profession for a long time and they know that. But the younger ones should be more involved in it as well. (Respondent 4, Hospital 1, FG T3)

Nurses mentioned it was essential to match and discuss the patients' wishes and needs with what the nurses considered important for the patient because some patients were not easily encouraged or motivated to mobilise specifically. Nurses rated these cases as challenging.

#### 5.2.2 Knowledge and Skills

During the implementation period, nurses reported some of their knowledge as insufficient (e.g., how to deal with unmotivated patients) and they noticed it could be refreshed or extended. Nurses of Hospital 2 mentioned the importance of knowledge and skills to deliver personalised nutritional and mobility care. Despite some insufficient knowledge, nurses of Hospital 2 indicated that personalised nutritional and mobility care was provided increasingly and on a daily basis during and at the end of the implementation period.

#### 5.2.3 Acceptability and Practicality in the Individual Factor

Findings revealed mixed opinions regarding nurses' evaluation of the acceptability and practicality of CR both during and at the end of the implementation period. During the implementation period, nurses mentioned a positive influence of CR on patients' recovery and CR was seen as an addition to usual care. Nurses of Hospital 2 indicated that CR served as a reminder and heightened awareness to deliver nutritional and mobility care:

I think awareness is already a very good step… we do not have to, we cannot always report everything, but at least it is in our minds that we are working on it and that we have highlighted it at least once during the shift. (Respondent 10, Hospital 2, FG T2)

However, despite the introduction of CR, most nurses still wished more awareness and intentional attention to nutritional and mobility care during the implementation period. At the end of the implementation period, nurses of Hospital 2 assessed CR as acceptable and practical because CR was part of usual care. These nurses considered CR valuable for new colleagues specifically because CR could provide structure and raise awareness. In contrast, nurses evaluated that CR was not completely performed by all nurses during and at the end of the implementation period. At the end of the implementation period, nurses rated CR as unimportant, experienced no differences between the period without and with CR and did not perform CR with awareness because nutritional and mobility care were integrated in usual care already. According to these nurses, CR did not noticeably change or add value to usual care, leading to an unclear evaluation of the acceptability and practicality of CR in this individual factor. Respondent 5 of Hospital 1 commented, ‘I find it strange that a structure needs to be imposed on daily tasks’ (FG T3). Another nurse stated:

… I actually don't notice a big difference… I feel like it's already part of a kind of daily routine. You don't really notice the difference between with or without comfort rounding… I'm not performing it with awareness, thinking, oh now I'm going to do a comfort rounding, or now I'm going to say something, for example… (Respondent 16, Hospital 2, FG T3)

### 5.3 Focus Group Interviews T1–T3: Social Factor

#### 5.3.1 Patient Characteristics

Patient characteristics either hindered or facilitated nutritional and mobility care and CR did not seem to have had any influence according to the nurses. Individual characteristics of patients included tiredness, fear, memory, patients' behaviour (e.g., patients' wait‐and‐see attitude or imperviousness to stimulus), isolation precautions and surgery‐related factors—such as dietary restrictions and presence of tubes and lines—limited patients' willingness, possibilities, or permissions for nutritional and mobility care. Also, opposite beliefs, wishes and needs between nurses and patients could result in struggles. Furthermore, talking too much about nutrition or mobility could actually be counterproductive:

…and I sometimes wonder about nutrition. Sometimes people say, ‘You talk about nutrition all the time, but I am really not hungry'… that it kind of works against it, so to speak and… that patients start to dig their heels in a bit more, that feeling. (Respondent 7, Hospital 2, FG T2)

In contrast, patients' preoperative fitness, motivation and positive experiences were facilitating mobility care.

#### 5.3.2 Nurse–Patient Relationship

During the implementation period, nurses of Hospital 2 indicated that building a relationship between a nurse and a patient was essential to perform CR. CR resulted in sharing mutual expectations with patients more often and nurses obtaining better insight into patients' preferences and nutritional and mobility status. Furthermore, nurses gained this insight from more patients than before and patients mobilised at an earlier moment.

### 5.4 Focus Group Interviews T1–T3: Organisational Factor

#### 5.4.1 Workload, Time Pressure and Team Culture

In the implementation period, nurses of both hospitals confirmed that workload and experienced time pressure were barriers for nutritional and mobility care and the performance of CR. This hindered the acceptability and practicality of CR. Specifically at Hospital 1, staff shortages and an increased workload and number of tasks were mentioned explanations. Furthermore, some nurses of Hospital 1 were not aware of CR during the implementation period due to perceived ineffective communication and some nurses did not perform CR because they had not adequately informed themselves about CR. Nurses of Hospital 2 experienced CR as time consuming, as an extra task and were searching for the best way to implement CR. Consequently, these nurses experienced that CR prevented them from doing other things and mentioned that CR could be skipped, forgotten, or not prioritised. Sometimes, the unpredictability of patient care made it difficult for nurses and patients to adhere to patients' individualised day structures or scheduled appointments regarding nutrition and mobility. This led to nurses who had to prioritise their work and had to make compromises that resulted in less patient participation, less information supply and support, less time to discuss with or respond to patients and a modified way or duration of mobilisation. During and at the end of the implementation period, nurses of Hospital 1 explained that their team culture and nurse‐colleagues' attitude were hindering the implementation of CR and hindered its acceptability, as it was the case with other implementations.

Because yes, it keeps being mentioned and everyone is nodding and agreeing. But in the end, the effect… it does not happen. (Respondent 4, Hospital 1, FG T2)

Despite a hindering team culture, nurses of Hospital 1 mentioned that they discussed nutrition and mobility routinely during physician's visits at the end of the implementation period, which suggested that CR may have had some positive impact on nutritional and mobility care.

#### 5.4.2 Design and Implementation

Only nurses of Hospital 2 mentioned that the design and implementation of CR influenced its acceptability and practicality. At first, some nurses evaluated the description of CR as ‘too extensive’:

When I see that form, I sometimes find it quite a lot to read at a glance what exactly is expected of me. So, I would sometimes really appreciate it if, for example, at 8:00 AM, I could see in three words what I need to discuss or at least something more organised, so to speak… (Respondent 8, Hospital 2, FG T1)

Nurses found the original CR design too rigid due to fixed times, which was a barrier. Despite repeated focus on this topic, not all nurses understood the CR during implementation. After tailoring the design to allow flexible timing, Hospital 2 nurses accepted CR more and reported improved performance. By the end, nurses felt sufficiently informed but found reminders too frequent and intrusive, sometimes perceiving them as pedantic or questioning their performance. Thus, tailoring improved CR's acceptability and practicality, though implementation still did not fully meet nurses' preferences.

#### 5.4.3 Reports, Registration and Handovers

Nurses' reports, electronic patient file entries and handovers were seen as essential and supportive for nutritional and mobility care delivery. They helped ensure continuity by increasing awareness and insight into patients' status and facilitated integration into daily routines and nurse communication—especially when problems or notable findings were present.

During implementation, nurses from both hospitals sometimes found reports and registrations to be incomplete, inconsistent and insufficiently informative—hindering insight into patients' nutritional intake and mobilisation, as well as continuity of care. At Hospital 1, this was mainly due to incompatibility between the electronic patient file and the nutrition registration system. At Hospital 2, a tailored smart phrase introduced after FG T1 helped raise awareness, improve reporting on nutritional and mobility status, support care evaluation and highlight comfort rounding.

I think so too. Especially with nutrition, you see those calories listed and then you think, I actually did not ask if it tasted good. So, it is just like with diuresis, but also with nutrition, you think, I need to go back and ask about that. (Respondent 10, Hospital 2, FG T2)

At the end of the implementation period, nurses of Hospital 2 stated that there were no insufficiencies in nurses' reports anymore.

### 5.5 Questionnaire

As demonstrated in Table 3, nurses of both hospitals provided attention to nutrition and mobility together with patients; performing CR was one of their priorities and respondents used the patient file when performing CR. Furthermore, nurses considered CR as of added value and helpful in providing structured attention to nutrition and mobility and this appeared to apply slightly more to nurses at Hospital 1 than at Hospital 2. Respondents at Hospital 1 were neutral towards the statement that there was sufficient time to perform CR, while respondents at Hospital 2 indicated that the time was sufficient. Respondents of both hospitals indicated that CR was often not performed in the day and evening shift (Table 4). Nevertheless, respondents of Hospital 1 indicated that all components of CR were performed most of the time and respondents of Hospital 2 mentioned that the components of CR were performed most of the time to very often, except the evaluation of care with patients (Table 4).

**TABLE 3:** Items 8.1–8.7 of the questionnaire: the attitude of nurses towards comfort rounding^a^.

Items	Hospital 1 median (IQR)^b^	Hospital 2 median (IQR)^b^	Hospital 1 and 2 median (IQR)^b^
	*n* = 16	*n* = 22	*n* = 38
8.1 Comfort rounding helps me in providing structured attention to nutrition and mobility	4 (3–4) *n* = 16	3 (2–4) *n* = 22	4 (3–4) *n* = 38
8.2 Together with the patient I provide attention to nutrition and mobility	4 (4–4) *n* = 16	4 (4–5) *n* = 22	4 (4–5) *n* = 38
8.3 The current implementation of comfort rounding match daily practice	3 (3–4) *n* = 16	3 (3–4) *n* = 22	3 (3–4) *n* = 38
8.4 Comfort rounding are not of added value for me	2 (1.5–2.5) *n* = 16 Reversed score: 4 (3.5–4.5)	3 (2–4) *n* = 21 Reversed score: 3 (2–4)	2 (2–3) *n* = 37 Reversed score: 4 (2.5–4)
8.5 I have sufficient time to perform comfort rounding	3 (2–3) *n* = 16	4 (3–4) *n* = 21	3 (3–4) *n* = 37
8.6 I use the patient file (EPIC/SAP) when I perform comfort rounding	4 (3–4) *n* = 15	4 (3–4) *n* = 22	4 (3–4) *n* = 37
8.7 Performing comfort rounding is not one of my priorities during my shift	2 (2–3) *n* = 16 Reversed score: 4 (3–4)	2 (2–3) *n* = 21 Reversed score: 4 (3–4)	2 (2–3) *n* = 37 Reversed score: 4 (2–4)

^a^ Answers on a five‐point Likert scale: 1 ‘strongly disagree’, 2 ‘disagree’, 3 ‘neutral’, 4 ‘agree’, 5 ‘strongly agree’.

^b^ Results shown in median Likert scores and interquartile range (IQR), measured according to Tukey's Hinges.

**TABLE 4:** Items 9–11.9 of the questionnaire: the execution of (several components of) comfort rounding by nurses^a^.

Items	Hospital 1 median (IQR)^b^	Hospital 2 median (IQR)^b^	Hospital 1 and 2 median (IQR)^b^
9. Think of the day shifts of the last 4 weeks, how often do you perform comfort rounding?	2 (2–2.5) *n* = 16	2 (2–2) *n* = 21	2 (2–2) *n* = 37
10. Think of the evening shifts of the last 4 weeks, how often do you perform comfort rounding?	3 (2–3) *n* = 14	2 (2–2) *n* = 20	2 (2–3) *n* = 34
11.1 I discuss the nutritional and mobility status with patients	4 (4–5) *n* = 16	5 (4–5) *n* = 21	5 (4–5) *n* = 37
11.2 I inform patients of the importance of nutrition and mobility	4 (4–5) *n* = 16	5 (4–5) *n* = 21	4 (4–5) *n* = 37
11.3 I support patients in the execution of nutritional and mobility interventions	4 (4–4) *n* = 16	5 (4–5) *n* = 21	4 (4–5) *n* = 37
11.4 I stimulate or motivate patients to eat/drink and move	4 (4–5) *n* = 16	5 (4–5) *n* = 21	4 (4–5) *n* = 37
11.5 I give advice and discuss possibilities/alternatives about nutrition and mobility with patients	4 (4–4.5) *n* = 16	4 (4–5) *n* = 21	4 (4–5) *n* = 37
11.6 I discuss patients' wishes, expectations and needs for nutrition and mobility	4 (3–4.5) *n* = 16	4 (4–4) *n* = 22	4 (4–4) *n* = 38
11.7 I make agreements with patients on nutritional intake and mobility	4 (4–4) *n* = 16	4 (4–5) *n* = 22	4 (4–5) *n* = 38
11.8 I execute the agreements made with patients about nutrition and mobility	4 (4–4.5) *n* = 16	4 (4–5) *n* = 22	4 (4–5) *n* = 38
11.9 I evaluate care related to nutrition and mobility with the patient	4 (4–5) *n* = 16	3.5 (3–4) *n* = 22	4 (3–4) *n* = 38

^a^ Answers on a five‐point Likert scale: 1 ‘never’, 2 ‘most of the time not’, 3 ‘as often as not’, 4 ‘most of the time’, 5 ‘very often/always’.

^b^ IQR = interquartile range, measured according to Tukey's Hinges.

### 5.6 Synthesis

Synthesis of the results demonstrates that nurses generally found CR to be acceptable and practical during the implementation period, as many of its elements aligned with existing care routines. It was regarded as a valuable addition that enhanced care delivery by raising awareness around nutrition and mobility, structuring attention to personalised care and deepening interactions with patients and families. However, some nurses felt CR added little to their current practice. Its performance varied and was not consistently carried out across shifts, partly due to differences in nurses' knowledge, skills and perceptions. Although most CR components were reportedly performed regularly, nurses acknowledged that CR was often not performed as intended during their shifts. This contradiction suggests that, while the content of CR was generally accepted and its components were performed, the concept of CR—with its fixed structure and design—was not always practical in everyday clinical practice. Nurses were divided on whether CR fit into their daily routines, with notable differences between hospitals. Team dynamics, rigid protocols and frequent reminders sometimes reduced motivation and perceived usefulness. Additionally, high workload, time pressure and unpredictable care demands were major barriers to perform CR as intended.

## 6 Discussion

The aim of this study was to evaluate nurses' perspectives on factors influencing the acceptability and practicality of CR, with a focus on personalised nutritional and mobility care. Data were collected through eight focus group interviews and were supported by data from a one‐time questionnaire.

On the one hand, results demonstrated that nurses perceived CR as acceptable and practical because it could enhance and improve the delivery of personalised nutritional and mobility care and most aspects of CR were already incorporated into and performed as part of usual care. On the other hand, low acceptability and practicality emerged, as nurses indicated that CR did not add value or change usual care. Nurses were divided on whether CR fit into their daily routines and although most aspects of CR were performed, it was not carried out as intended. The results regarding CR's acceptability and practicality are therefore contradictory.

Comparison with previous studies demonstrated similar contradicting results regarding CR's acceptability and practicality. CR could be seen as acceptable as nurses indicated that CR could support the delivery of comprehensive and consistent care to patients, could have added value to improve quality of care (Christiansen et al. 2018; Leamy et al. 2023; Ryan et al. 2019) and could inform and alert new colleagues in particular to perform fundamental patient care (Leamy et al. 2023; Ryan et al. 2019). However, previous research also demonstrated that roundings were not always applied to daily practice, were not consistently performed during allocated ‘rounding’ time and were integrated into other patient care activities (Leamy et al. 2023), indicating a low acceptability and practicality of CR.

A possible explanation for the low acceptability of CR could be found in the absence of a clear necessity to change usual personalised nutritional and mobility care into CR (Christiansen et al. 2018), as nurses in this study reported performing most components of CR in usual care already and did not prefer a new structure to deliver personalised nutritional and mobility care. Another explanation for the low acceptability of CR could be found in the negative influence of CR on nurses' autonomy. Previous studies revealed that implementing CR could hinder nurses' professional autonomy if prescriptive and restrictive protocols prevent nurses from personalising their care or critically thinking (Christiansen et al. 2018; Ryan et al. 2019). Conversely, flexible and adaptive protocols regarding CR facilitate its implementation over time (Christiansen et al. 2018) and could positively influence the acceptability and practicality of CR. This was demonstrated at hospital 2, where the attitude to and performance of CR improved after tailoring to a more flexible design, although the questionnaire revealed that CR was still often not performed as intended by nurses.

Differences between hospitals—such as nurse–patient ratios, staff shortages and perceived workload—may have contributed to the contrasting results as well. Prior research demonstrated that CR compliance and effectiveness were hindered, among others, by time constraints, workload, competing priorities, staffing issues and difficulty integrating CR into daily routines (Christiansen et al. 2018; Leamy et al. 2023). Hospital 1 faced more challenges than Hospital 2 due to higher nurse–patient ratios, perceived lack of time, workload and a restrictive team culture. Surprisingly, nurses at Hospital 1 still saw slightly more added value in CR, though they noted few changes during the implementation period. Hospital 2, after tailoring CR, observed improved performance and increased patient participation. Despite these differences, no clear variation in CR's overall performance, acceptability, or practicality was found. This suggests that a positive attitude alone does not lead to behavioural change. Acceptability and practicality appear context‐dependent and not generalisable. Future implementation should consider both positive expectations and barriers such as workload and team culture, while learning from the success of Hospital 2 in tailoring CR to practice.

In this feasibility study, evaluating the acceptability and practicality of CR is essential to determine its suitability for further investigation. Prior to initiating a full‐scale study, it is important to carefully consider the positive and negative factors that influence CR's acceptability and practicality. A prerequisite for further testing is that nurses perceive a clear need to shift from conventional nutritional and mobility care practices to the CR approach. Additionally, it is crucial that the design of CR is tailored to align with current nursing practices and respects nurses' preferences for professional autonomy. This requires a careful co‐design process involving a representative group of nurses from the participating ward. Finally, organisational support is required to enable the implementation and performance of CR, as factors such as workload, time constraints and team culture have played a key role in the delivery of CR.

### 6.1 Strengths and Limitations of the Work

Strengths of this study were found in the study design. CR were tested at two university medical centres for 1 year and nurses were interviewed before, during and at the end of the implementation period. The qualitative design fostered a broad and in‐depth knowledge about nurses' experiences regarding CR and the heterogeneity between the two hospitals may signify a difference in the role that CR could play in patient care. Furthermore, the design facilitated enough time for the researchers to inform, educate and interview nurses and resulted in enough time for nurses to experiment with CR and to discuss their experiences in the FGs or questionnaire. The quantitative results of the questionnaire supported the findings of the focus group interviews by capturing the perspectives of as many nurses as possible from both departments.

Although a predominantly qualitative approach was deliberately chosen during the design phase to assess the acceptability and practicality of CR from nurses' perspectives, a more quantitative design could also have been selected, for example by using the Theoretical Framework of Acceptability (TFA) questionnaire. Using the TFA allows for standardised data collection across time points and provides an efficient means of gaining insight into potential challenges nurses may experience regarding the acceptability of CR (Sekhon et al. 2022). To our knowledge, this is the first study that investigated the acceptability and practicality of CR with attention specifically to nutritional and mobility care and patient participation. This study offered the opportunity to investigate if CR could contribute to the performance of fundamental nursing care (Kitson 2018). Prior literature demonstrated similar results regarding the acceptability and practicality of CR, although the content of the roundings was not similar. Given the substantial overlap in findings, it seems that the specific content of CR may not be the main factor influencing its acceptability and practicality. Instead, this study highlighted that the structure and flexibility of rounding in usual care seem to matter more than the exact details discussed during rounds. Some limitations were found in the role of the researcher of Hospital 2, differences in educational levels between respondents of the two hospitals and the challenge at both hospitals to recruit participants for the FG. The researcher from Hospital 2 (FB) worked as a clinical academic nurse, combining bedside nursing care with nursing science. She was present during the FGs at Hospital 2. This could have led to participant bias, where participants gave socially desirable answers or confirmation bias, where the researcher interpreted results in favour of her hypothesis. To minimise bias and enhance methodological rigour, member checking, double coding and involvement of independent researchers were implemented and were therefore strengths of this study. Another limitation was the varying educational levels of the nurses who completed the questionnaire. This led to less homogeneous settings and less clear generalisability of the results. Also, the challenge to recruit nurses to participate in the focus group interviews led to convenience sampling with a minimum of participants in each focus group interview. The heterogeneity and convenience sampling in this study might have biased the results.

### 6.2 Recommendations for Further Research

A different approach to personalised nutritional and mobility care is needed, as nutritional and mobility care are vulnerable to being left undone. This study highlighted that a fixed structure is not desired, that nurses' performance of nutritional and mobility care varies and remains too dependent on the individual knowledge and skills of nurses. Future research should investigate effective approaches for the consistent delivery of high‐quality, personalised nutritional and mobility care, without too fixed structures, allowing for nurses' autonomy and regardless of which nurse is providing the care. A Delphi study involving multiple nurses from different hospitals could help address this question. Despite tasks and responsibilities being similar across educational levels, future research could explore whether differences in nurses' education influence attitudes towards personalised nutritional and mobility care delivery and comfort rounding, as well as whether these differences lead to varying needs for tailoring such care and which approaches are most effective for each group in achieving personalised care. Furthermore, nurse managers' perspectives were not examined in this study; however, it is essential that future research incorporates their views when developing and implementing structured approaches to personalised nutritional and mobility care. Their insights can drive organisational change and support the successful adoption of interventions. Moreover, the effects of personalised nutritional and mobility care on patients' nutritional or mobilisation status and patients' experienced participation are still unknown. Future research should investigate if personalised nutritional and mobility care and patient participation affect patient outcomes, such as nutritional and mobility status, satisfaction, or self‐care. If a positive trend becomes visible, it serves as evidence and an extra incentive for nurses to develop and organise their care in a way that enables them to provide this personalised care as well.

### 6.3 Implications for Policy and Practice

Before implementing CR, it is important to consider several key points. First, determine if there is a need to change the usual care. Second, CR needs to be tailored to the specific setting where it is implemented to allow nurses flexibility in their shifts and to personalise their care to their patients' needs. Finally, the team must be prepared and ready to undertake such an implementation, ensuring that all nurses make a concerted effort towards achieving a successful implementation.

## 7 Conclusion

CR is acceptable and practical in daily practice when flexibility in execution is allowed because nurses value its importance and usefulness to improve attention for patients' nutritional and mobility care and patient participation. Individual, social and organisational factors included both facilitators and barriers to the acceptability and practicality of CR. When components of CR were already incorporated in usual care and performed by nurses, nurses were resistant to implementing a fixed schedule to perform this aspect of usual care, which hindered CR's acceptability and practicality. Although nurses did not consider the CR concept crucial, the implementation period led to notable improvements in nutritional and mobility care as well as patient participation, according to the nurses.

## 8 Implications for the Profession and/or Patient Care

Comfort roundings' concept does not align well with current nursing practice. Greater tailoring to nurses' preferences or alternative approaches to structuring personalised nutritional and mobility care are recommended.

## Author Contributions

All authors made substantial contributions to conception and design, or acquisition of data, or analysis and interpretation of data. Given final approval of the version to be published. Each author should have participated sufficiently in the work to take public responsibility for appropriate portions of the content. Agreed to be accountable for all aspects of the work in ensuring that questions related to the accuracy or integrity of any part of the work are appropriately investigated and resolved. Involved in drafting the manuscript or revising it critically for important intellectual content: Femke Becking‐Verhaar, Harm Van Noort, Marian De Van Der Schueren, Hester Vermeulen, Getty Huisman‐De Waal.

## Funding

This work was supported by the Academische Alliantie of Maastricht University Medical Center+ and Radboud University Medical Center, PI‐00423.

## Disclosure

Declaration regarding the used statistics: As only descriptive statistics were used, the authors agree to take responsibility for ensuring that the choice of statistical approach is appropriate and is conducted and interpreted correctly as a condition to submit to the Journal.

## Conflicts of Interest

The authors declare no conflicts of interest.

## Supporting information


Appendix A.

Appendix B.

Appendix C.

Appendix D.

Appendix E.

Appendix F.

## Data Availability

Data available in article Supporting Information.
