# Supplementary material for: Evaluating Nurses' Perspectives on the Acceptability and Practicality of Comfort Rounding for Personalised Nutritional and Mobility Care in Surgical Wards: A Mixed‐Methods Feasibility Study
Source: J Adv Nurs. 2025 Dec 21;82(8):8158–71. doi: 10.1111/jan.70462 (PMC13356403; doi:10.1111/jan.70462)
Supplement: Supplementary file 1 — Appendix A. [file JAN-82-8158-s003.docx]

**Appendix A. Structure and content of comfort rounding**

Schedule of comfort rounding at Hospital 1 in day shift and evening shift

| **Day shift** | | |
| --- | --- | --- |
| **Time** | **Nutrition** | **Mobility** |
| 7:15 – 9:00 |  | - Discuss wishes and possibilities regarding ADL (Activities of Daily Living) and mobilization in the morning. - Provide advice on alternatives/options. - Discuss expectations and make agreements |
| 8:30 – 9:00 | - Patient is offered breakfast. | - Have the patient sit in the chair during breakfast and provide support if needed. |
| 10.00 – 12.00 | - Inquire about food intake compared to normal: - More than usual - Unchanged - Less than usual - Ask about: - Appetite - Taste - Nausea/vomiting - Swallowing difficulties - Sore mouth - Constipation/diarrhea - Feeling full quickly - If there are problems with food intake: - Explain the (possible) cause - Discuss wishes/possibilities - Provide advice on alternatives/options - Discuss expectations and make agreements (and follow through) | - Discuss with the patient how their mobility is and where support is needed: - Use of aids - Moving in bed - Moving from lying on the back to sitting on the edge of the bed - Moving from bed to chair - Standing up from a chair - Walking in the hospital room - Provide support as needed based on previous agreements. |
| 12:00 – 12:30 | - Patient is offered lunch. | - Have the patient sit in the chair during lunch. |
| 13.00 – 14.00 | - Rest hour for patients. | - Rest hour for patients. |
| 14.00 – 15.00 | - Check the food list (if agreed upon). - Evaluate expectations and agreements made, and adjust them if necessary, in consultation with the patient. - Report in SBARR/ADL. | - Evaluate expectations and agreements made, and adjust them if necessary, in consultation with the patient. - Provide support as needed based on agreements made. - Report in SBARR/ADL. |
| 15.00 – 15.45 | - Discuss food/fluid intake and expectations/agreements made during bedside handover. | - Discuss mobility and expectations/agreements made during bedside handover. |

| **Evening shift** | | |
| --- | --- | --- |
| 15.00 – 15.45 | - Discuss food/fluid intake and expectations/agreements made during bedside handover. | - Discuss mobility and expectations/agreements made during bedside handover. |
| 17.00 – 18.00 | - Patient is offered dinner. | - Have the patient sit in the chair during dinner and provide support if needed. |
| 18.00 – 22.00 | - Check the food list (if agreed upon). - Evaluate with the patient how eating and drinking went today: - More than usual - Unchanged - Less than usual - Ask about: - Appetite - Taste - Nausea/vomiting - Swallowing difficulties - Sore mouth - Constipation/diarrhea - Feeling full quickly - If there are problems with food intake: - Explain the (possible) cause - Discuss wishes/possibilities - Provide advice on alternatives/options - Report in SBARR/ADL. | - Provide support as needed based on agreements made. - Evaluate with the patient how their mobility has been today: - Use of aids - Moving in bed - Moving from lying on the back to sitting on the edge of the bed - Moving from bed to chair - Standing up from a chair - Walking in the hospital room - Report in SBARR/ADL. |

Schedule of comfort rounding at Hospital 2 in day shift and evening shift

| **Time** | **Nutrition** | **Mobility** |
| --- | --- | --- |
| 8.00 | - Inform the patient how many calories and proteins they consumed yesterday, when closing the fluid balance and food registration. - Inform the patient how many calories and proteins they need for recovery, and what they should ideally aim for during their stay. | - Discuss with the patient their wishes and possibilities regarding mobilisation in the morning (including ADL - Activities of Daily Living). |
| 9.00 – 11.30 | - Discuss mutual expectations regarding food intake with the patient. - Check if the patient is familiar with the FoodforCare concept; if not, ask the nutrition assistants to explain it. - Consult with the patient on their food intake goals for the day and provide nursing advice accordingly. - Inform the patient about proteins (awareness: why they are needed, where proteins are found). - Coordinate with the patient if and when they need help making choices about food or with eating and drinking themselves   *If patients do not eat and drink sufficiently:*   - Discuss with the patient the reasons why they are not eating and drinking enough. - Inform and discuss alternatives or possible solutions with the patient (different food products, (other) nutritional drinks, anti-emetics, food from home). | - Carry out what was agreed upon with the patient in the morning (e.g., washing at the edge of the bed, showering, sitting in the chair) |
| 12.00 –  15.00 | - Provide support during meals if necessary (consider the position and place where the patient eats). - Evaluate with the patient how eating and drinking went during breakfast and lunch. - Inform the patient about how many calories and proteins they have consumed so far. - Adjust previously made expectations in consultation with the patient, if necessary | - Check if the agreements made this morning regarding mobilisation are still valid/feasible. - Discuss with the patient their wishes and possibilities regarding mobilisation in the afternoon (consider 'eating at the table' and mobilising with family, for example). - Carry out what was agreed upon with the patient in the afternoon. |
| 17.00 | - Provide support during meals if necessary (consider the position and place where the patient eats). | - Discuss with the patient their wishes and possibilities regarding mobilization in the evening (include ADL such as brushing teeth, for example), and carry this out |
| 20.00 – 22.00 | - Evaluate with the patient how eating and drinking went today. - Inform the patient about how many calories and proteins they have consumed today | - Carry out what was agreed upon with the patient at 17:00. - Evaluate with the patient how their mobilization went today. |
