# Supplementary material for: Evaluating Nurses' Perspectives on the Acceptability and Practicality of Comfort Rounding for Personalised Nutritional and Mobility Care in Surgical Wards: A Mixed‐Methods Feasibility Study
Source: J Adv Nurs. 2025 Dec 21;82(8):8158–71. doi: 10.1111/jan.70462 (PMC13356403; doi:10.1111/jan.70462)
Supplement: Supplementary file 2 — Appendix B. [file JAN-82-8158-s006.pdf]

JAN
Leading Global Nursing Research

Evaluating nurses' perspectives on the acceptability and practicality of comfort rounding for personalised nutritional and mobility care in surgical wards: a mixed-methods feasibility study

Journal:	*Journal of Advanced Nursing*
Manuscript ID	JAN-2025-2621.R2
Wiley - Manuscript type:	Feasibility Study (Direct Via EEO)
Keywords:	Nurse - Patient Relationships, Nutrition, Nurse - Patient interaction, Nurse roles, Organisational Behaviour, Patient safety, Post-operative Care, Surgical Nursing
Category:	Nursing

SCHOLARONE™
Manuscripts

Title

Evaluating nurses' perspectives on the acceptability and practicality of comfort rounding for personalised nutritional and mobility care in surgical wards: a mixed-methods feasibility study

Running title

Acceptability and practicality of CR

Authors

Femke BECKING-VERHAAR[1], Fanny PELZER[2], Harm VAN NOORT[1], Cariline ROOSEN[1], Yvonne VAN EIJK-HUSTINGS[3], Marian DE VAN DER SCHUEREN[5], Hester VERMEULEN[4] and Getty HUISMAN-DE WAAL[1,4]

[1]Department of surgery, Radboud University Medical Centre, Nijmegen, The Netherlands

[2] Department of Nursing, Maastricht University Medical Centre, Maastricht, The Netherlands

[3]Department of Clinical Epidemiology and Medical Technology Assessment (KEMTA), Maastricht University Medical Centre, The Netherlands

[4] IQ Health, Radboud University Medical Centre, Nijmegen, The Netherlands

[5] HAN University of Applied Sciences, Nijmegen, The Netherlands; Wageningen University and Research, Wageningen, The Netherlands

Review Copy

Author contributions

Criteria	Author Initials
Made substantial contributions to conception and design, or acquisition of data, or analysis and interpretation of data;	FBV, FP, HVN, CR, YVEH, MDVDS, HV, GHDW

Involved in drafting the manuscript or revising it critically for important intellectual content;	FBV, HVN, MDVDS, HV, GHDW
Given final approval of the version to be published. Each author should have participated sufficiently in the work to take public responsibility for appropriate portions of the content;	FBV, FP, HVN, CR, YVEH, MDVDS, HV, GHDW
Agreed to be accountable for all aspects of the work in ensuring that questions related to the accuracy or integrity of any part of the work are appropriately investigated and resolved.	FBV, FP, HVN, CR, YVEH, MDVDS, HV, GHDW

Acknowledgments

Special thanks to Wilmieke Bahlman-van Ooijen, Annemarie Sänger, Lloyd Brandts, and students of the Bachelor of Nursing. Artificial intelligence was used to translate or correct some sentences from the authors' language into English, without changing the content.

Funding Statement

This study was funded by the Academic Alliance Fund of Radboud university medical center and Maastricht University Medical Center+.

Conflict of interest statement

The authors declare no conflict of interest

Declaration regarding the used statistics

As only descriptive statistics was used, the authors agree to take responsibility for ensuring that the choice of statistical approach is appropriate and is conducted and interpreted correctly as a condition to submit to the Journal.

Corresponding author

Review Copy

femke.becking-verhaar@radboudumc.nl, 0031-(0)243613438

Evaluating nurses' perspectives on the acceptability and practicality of comfort rounding for personalised nutritional and mobility care in surgical wards: a mixed-methods feasibility study

Aim: To evaluate nurses' perspectives on factors influencing the acceptability and practicality of comfort rounding, focussing on personalised nutritional and mobility care.

Design: Mixed-methods feasibility study

Methods: Focus group interviews with nurses were conducted before, during, and at the end of the implementation period (2022-2023). A questionnaire assessed acceptability and practicality among nurses at the end of the implementation. Data were analysed using directed content analyses and descriptive statistics.

Results: Comfort rounding's acceptability and practicality were influenced by nurses' attitudes, knowledge and skills, patient characteristics, and the nurse-patient relationship. Barriers included workload, time pressure, team culture, and the extensive, rigid design of comfort rounding. Questionnaire responses demonstrated nurses perceived added value of comfort rounding and frequently engaged patients in activities related to nutrition and mobility. However, it was not performed as originally intended.

Conclusion: Nurses considered personalised nutritional and mobility care important and frequently provided it during 'usual care'. However, nurses were critical of comfort rounding's acceptability and practicality and did not perform it as intended.

Implications for the profession and/or patient care:

Comfort roundings' concept does not align well with current nursing practice. Greater tailoring to nurses' preferences or alternative approaches to structuring personalised nutritional and mobility care are recommended.

Acceptability and practicality of CR

Impact:

- **What problem did the study address:**
  - Hospitalised patients often receive suboptimal nutritional care and are largely inactive.
  - The challenge is to integrate personalised nutritional and mobility care effectively into standard nursing practice to enhance patient safety and well-being.
  - Comfort rounding could improve patient safety and satisfaction; however, there is no research evaluating the feasibility of comfort rounding in relation to personalised nutritional and mobility care.

- **What were the main findings:**
  - Comfort rounding was generally perceived as valuable and aligned with existing care routines, but its rigid structure was often considered impractical.
  - Comfort rounding was not performed as originally intended, due to the influence of individual, social, and organisational factors.
  - Flexibility in execution emerged as critical factor for successful integration.

- **Where and on whom will the research have an impact?**
  - Comfort rounding can enhance attention to nutrition, mobility, and patient participation when adapted to local contexts and delivered with flexibility.
  - Policymakers and nurse leaders should avoid rigid protocols and instead support tailored implementation strategies alongside the practical delivery of locally tailored interventions.

Reporting Method: Consolidated criteria for reporting qualitative research and Checklist for Reporting of Survey studies

Patient or Public Contribution: Nurses were involved in all stages of the study, contributing through focus group interviews and completing a questionnaire to help develop and evaluate comfort rounding.

Review Copy

Keywords: comfort rounding, intentional rounding, hourly rounding, nutrition, nutritional care, mobilisation, mobility care, patient participation, fundamental care, qualitative research

What does this paper contribute to the wider global clinical community?

- Comfort rounding may support fundamental care delivery
- Acceptability and practicality of comfort rounding depend on flexibility

Trial and protocol registration: PaNaMa Research Management System, number 112832

Review Copy

1. Introduction

According to the Fundamentals of Care framework (FoC), nurses are in a key position to strive for safe, effective, and high-quality care within the physical, psychosocial, and relational domain incorporating nutritional and mobility care and patient participation (Kitson, 2018; Mitchell, 2008). Several studies demonstrated that attention for early nutrition and mobilisation, as well patient participation, could reduce the risk of post-surgical complications and positively affect patients' recovery (Koenders et al., 2021; Koenders et al., 2020; Ljungqvist & Hubner, 2018; van den Berg et al., 2021). Furthermore, it could enhance patients' psychological and social well-being (Kalisch et al., 2014; Koenders et al., 2021; Rios et al., 2021). However, nutritional and mobility care are vulnerable to care left undone (van Belle et al., 2020; Van Den Berg, Vermeulen, et al., 2023), hospitalised patients sometimes receive suboptimal nutritional care (Ten Cate et al., 2021), and hospitalised patients remain largely inactive (Fazio et al., 2020). So, the challenge is to integrate nutritional and mobility care and patient participation more effectively into standard nursing practice to enhance patient safety and well-being. This could be achieved through comfort rounding (CR)—predefined and structured rounds where nurses visit their patients with a focus on nutrition, mobility, and patient participation on predetermined time intervals and with a standardised protocol during their shifts (Harris et al., 2019; Sims et al., 2018). This study evaluates the acceptability and practicality of CR from nurses' perspectives.

2. Background

Recovery from major abdominal surgery is challenging due to complications which occur in 30-40% of the patients. Complications are the main cause of high morbidity and mortality, prolonged hospital stays, and reduced life expectancy (Ferreyra et al., 2009; Jakobson et al., 2014; Khuri et al., 2005; Veličković et al., 2020).

Studies have demonstrated that approximately 10% of surgical patients are malnourished (Kruizenga et al., 2016; Ten Cate et al., 2021), and hospitalised patients often receive suboptimal nutritional care due to competing priorities and ward regulations for example (Ten Cate et al., 2021). Furthermore, evidence indicates that hospitalised patients remain largely inactive, with sedentary behaviour accounting for 87% to 100% of their day (Fazio et al., 2020). These factors adversely affect recovery after surgery. The Enhanced recovery after

surgery (ERAS) guidelines aim to improve recovery by reducing perioperative stress responses and enhancing postoperative outcomes. Within the multidisciplinary team—including surgeons, anaesthesiologists, and nurses—nurses play a pivotal role throughout the preoperative and postoperative phases (Ljungqvist & Hubner, 2018; Visioni et al., 2018). In particular, during the postoperative phase, nurses are essential in reducing complications and promoting recovery by prioritising early nutrition and mobilisation, both key components of the ERAS approach (Ljungqvist & Hubner, 2018). Early nutrition and mobilisation contribute significantly to enhanced recovery: lowering the incidence of physical complications and mortality following surgery (Correia & Waitzberg, 2003; EPUAP et al., 2019; Nydahl et al., 2023; van der Leeden et al., 2016), while also positively impacting patients' psychological and social well-being (Kalisch et al., 2014; Koenders et al., 2021; Rios et al., 2021) and functional capacity postoperatively (de Almeida et al., 2017). Collectively, these findings underscore the indispensable role of nurses in delivering effective nutritional and mobilisation care to support surgical recovery.

According to the Fundamentals of Care framework (FoC), nurses are in a key position to strive for safe, effective, and high-quality care within the physical, psychosocial, and relational domain (Kitson, 2018; Mitchell, 2008). Attention for early nutrition and mobilisation is encompassed in the physical domain of the FoC framework (Feo et al., 2018; Feo et al., 2017; Kitson, 2018) and could be supported by activities in the psychosocial and relational domain, incorporating patient participation. Patient participation is defined as an interactional process between nurses and patients, in which mutual negotiation and patients gaining insight through consideration are the core elements (Larsson et al., 2007; Sahlsten et al., 2007). Patient participation can enhance nutritional care by identifying patients' eating difficulties and dietary preferences (van den Berg et al., 2021), and individual patient education and goal setting could lead to improved physical performance and body weight of patients (van den Berg, de van der Schueren, et al., 2023). Furthermore, patient participation can improve mobility care by discussing, setting, and achieving physical activity goals through tailored interventions that consider patients' mobility needs and preferences (Koenders et al., 2021; Koenders et al., 2020). Despite the aforementioned importance of nurses actively approaching and involving patients across all domains of FoC, nurses still tend to focus on task-oriented activities primarily within the physical domain, which reflects

nurses' biomedical focus. Furthermore, it is not always evident that nurses consistently incorporate patients' needs and experiences, engage in discussions about care planning, or actively facilitate patient participation in these processes (van Belle et al., 2020; Van Den Berg et al., 2023). Moreover, patient mobilisation and certain aspects of nutritional care are often at risk of being left undone (Griffiths et al., 2018). Patients themselves also do not always take the initiative, due to a lack of awareness about the risks of malnutrition or uncertainty regarding mobilisation, which can lead to physical inactivity and highlights the need for professional support (Koenders et al., 2020; Van Den Berg, Vermeulen, et al., 2023). Therefore, a key challenge is to systematically embed nutritional and mobility care and patient participation structurally into the organisation of daily nursing practice. To this end, CR has emerged as a possible solution, with specific attention to nutrition, mobility, and patient participation. Studies have demonstrated that CR can enhance patient safety (e.g., fall prevention) and patient satisfaction (Daniels, 2016; Di Massimo et al., 2022), as well as reduce nurses' workload and foster a more supportive and efficient work environment (Ryan et al., 2019).

Review Copy

3. The Study

3.1 Aim

In this study, CR with structured focus on nutrition, mobility, and patient participation is developed, tailored, and implemented within the organisation of nursing practice on two surgical wards in two university hospitals for a one-year period. The implementation is guided by an implementation framework (Grol et al., 2013). This study aims to evaluate nurses' perspectives on the acceptability and practicality of CR, with specific attention to nutrition, mobility, and patient participation, in nursing practice.

4. Methods

4.1 Design

In this mixed-methods feasibility study, the focus was on to evaluate whether CR can and does work in nursing practice and to discover barriers and facilitators. Therefore, two areas

of focus were studied as primary outcome: the 'acceptability' and 'practicality' of CR (Bowen et al., 2009). Evaluating acceptability would demonstrate nurses' reactions to CR, its appropriateness and fit in daily nursing practice, nurses' satisfaction with CR, and their willingness to use CR. Evaluating practicality would reveal how easily CR could be executed by nurses and which facilitating and hindering factors are contributing to this (Bowen et al., 2009). Focus group interviews (FGs) were used to evaluate nurses' experiences and were held at four moments (see Table 1). This design allowed for data comparison across baseline and follow-up measurements and to determine if slight differences appeared over time. After FG T3, an expert-based questionnaire was distributed to all nurses. This questionnaire was used as a supplementary tool alongside the FGs and aimed to evaluate the acceptability and practicality of CR among a larger group of nurses. Therefore, the results of the questionnaire were used to complement the findings from the FGs. Consolidated criteria for reporting qualitative research (COREQ) were used for reporting the qualitative part of the study (Tong et al., 2007) (see Appendix E) and the Checklist for Reporting of Survey studies was used to report the questionnaire study (Sharma et al., 2021) (see Appendix F).

[Insert Table 1. Timeline of data collection and activities regarding the study]

N Copy

4.2 Study setting

The study spanned from September 2021 to September 2023, with CR implemented for one year on two surgical wards in two university hospitals in The Netherlands. The first ward comprised 40 beds, 35 qualified and registered nurses, either vocationally or bachelor-educated (numbers of each not registered), 3 clinical team leaders, and patients were mainly admitted for abdominal surgery with underlying gastroenterological, oncological, or gynaecological diseases (e.g., colorectal surgery, debulking, and uterus extirpation). Nurse-patient ratio in the day shift was 1:8 and in the evening shift 1:12. The used electronic patient file was Oracle Cerner (www.cerner.com). The ward of this hospital is referred to as 'Hospital 1'. The second ward, referred to as 'Hospital 2' comprised 18 beds, 33 qualified and registered nurses—11 vocationally educated and 22 holding a bachelor's degree—2 clinical team leaders, and patients were mainly admitted for elective major abdominal-oncological surgery (e.g., Hyperthermic Intraperitoneal Chemotherapy, pancreaticoduodenectomy, esophagectomy, hemi hepatectomy, abdominal perineal resection). Nurse-patient ratio in

the day shift was 1:3 and in the evening shift 1:6. The used electronic patient file was EPIC (www.epic.com). In both hospitals, there were no differences in care activities or responsibilities between vocationally-educated nurses and those with a bachelor's degree during the study period. Furthermore, both educational levels shared the same statutory registration in the national register for healthcare professionals at that time. Thus, all nurses worked within a single team without clear demarcation of responsibilities.

Both hospitals were equipped with allied health professionals such as physiotherapists and dieticians. Although both centres are university hospitals, there are differences in the nurse-patient ratio. Multiple factors may be involved. One possible explanation for the variation in nurse-patient ratio is the difference in surgical procedures and complexity. The participating department at Hospital 1 performs relatively more colorectal surgeries, which are often considered less complex than the extensive pancreatic and liver surgeries more commonly performed at Hospital 2. Additionally, the determination of staffing ratios was also influenced by the internal organisational structures of the individual hospitals.

Review Copy

4.3 Sampling

A number of five to eight participants was targeted for each focus group interview. Nurses of both hospitals were invited by e-mail to participate in the FGs when they met inclusion criteria. Reminders were sent after one week each time. A convenience sampling approach was used: all nurses who voluntarily chose to participate were able to do so, in order to obtain a broad range of experiences within the heterogeneous groups of nurses. Nurses willing to participate provided informed consent before the FG. At T3, all nurses from both hospital wards who met inclusion criteria were invited to voluntarily complete a one-time anonymous questionnaire. A convenience sampling method was used for the questionnaire as well, striving for as many as possible respondents.

4.4 Inclusion criteria

For the FGs and questionnaire, similar including criteria for nurses applied:

- Graduate nurses working on regular base on the specific ward
- Nurses with ≥ 3 months' work-experience on the specific ward
- Nurses working in direct patient-related care

4.5 Study intervention: Development and implementation of comfort rounding

CR refers to structured visits (rounds) nurses make to patients at set times, following a standardised protocol (Sims et al., 2018). In this study, CR was developed and implemented at both surgical wards and meant that nurses would speak regularly with their patients about mobilisation and nutrition (e.g. the importance of both to recover from surgery, support needed) and how patients wanted to participate. This process was structured by the implementation strategy of Grol et al. (Grol et al., 2013) and included the phases orientation, insight, acceptance, change, and sustainability. These phases guided the implementation activities as described in the row 'Activities' in Table 1. Although this implementation strategy was used to guide the introduction of CR on both wards, this study was explicitly not classified as implementation research. Before proceeding with full implementation, the aim of this study was to first explore the acceptability and practicality of CR.

At study start, each hospital developed the content and structure of CR for both day and evening shifts, based on feedback from FG T0 (see Appendix A). The structure of CR consisted of time blocks during which nurses were expected to talk with patients about certain topics or take specific actions related to mobility or nutrition. CR was continuously tailored in both settings to fit the needs and possibilities of the particular context and tailoring was guided by findings from FG T1 and T2 (see Table 2). In comparison, usual care in both hospitals involved regular rounds during which vital signs were measured, fluid balances were recorded, and medication was administered, but structured attention (i.e., at predetermined time intervals and following a standardised protocol) to nutritional intake, mobility, and patient participation related to these aspects, as defined to the definition of patient participation, was not present yet.

[Insert Table 2. Development and tailoring of comfort rounding (CR)]

4.6 Data collection

FGs offered the possibility to collect a range of nurses' perceptions and points of view about CR in a limited period through group interactions and discussions (Krueger, 2014; Morgan, 1996). Therefore, eight FGs with nurses were held (see Table 1). FG results reflected the views of a limited subset of the total number of nurses. To gather comprehensive feedback

Review Copy

on nurses' CR performance and the acceptability and practicality of CR among a larger group of nurses, an expert-based questionnaire was distributed to all nurses of both wards after FG T3 (see Table 1). Since it was clear beforehand that there were no differences in care activities or responsibilities between vocationally-educated nurses and nurses with a bachelor's degree during the study period, it was decided not to distinguish between educational levels when composing the participants for the focus group interviews, distributing the questionnaires, or analysing the data.

Focus group interviews

An interview guide and two semi-structured topic lists, one for the baseline (T0) and one for the intermediate and follow-up measurements (T1–T3), were constructed by female researchers FB, FP, GH and YvE. Both lists were based on four factors influencing the implementation of an innovation: individual, social, organisational, and societal factors (Wensing et al., 2013a) (Appendix B). The topics encompassed how nurses performed nutritional and mobility care, how they involved patients, and specific questions about CR, which varied between the topic lists of T0 and T1-T3. At T0, nurses were asked about their perceptions and assessments of the forthcoming CR implementation. At T1–T3, discussions focused, among other things, on the introduction and execution of CR by nurses. FGs in both hospitals were conducted in quiet locations in the hospital, audio-recorded, and transcribed verbatim. They were moderated by female early-career researchers FP (nurse practitioner, MANP) and FB (nurse scientist, PhD candidate), who were trained in qualitative methods, and by trained and supervised female nursing students. Only the researchers and participants were present. Field notes were sometimes, so not structurally taken. Data were pseudonymised, and transcripts were shared with participants for member checking to ensure accurate representation of nurses' perspectives. The participants had no feedback on the transcripts.

Questionnaire

A 27-item questionnaire was made by six experts in the domain of nursing care (YvE, HvN, FP, GH, FB, AS) and one statistician (LB). See Appendix C.

The first six questions gathered respondent characteristics. Question seven asked if respondents were familiar with CR; if so, respondents were asked to answer questions eight

Review Copy

to eleven. Question eight and eleven consisted of seven respectively nine statements. Questions nine and ten asked how often CR were performed. Questions nine to eleven were answered using a five-point Likert scale, ranging from 1 ('totally disagree' or 'never') to 5 ('totally agree' or 'always'). Questions 8.4 and 8.7 were negatively formulated, the answers of these questions were reverse-coded because a higher score on each question indicated a more positive attitude to CR. If respondents were not familiar with CR, they were asked to respond to the open-ended questions twelve and thirteen to support the researchers with additional information. Question twelve and thirteen were excluded from this study as they did not contribute to assessing the acceptability and practicality of CR. The questionnaire was administered on paper, for two weeks. Data were processed anonymously.

Review Copy

Data saturation

As this was a feasibility study designed to generate initial insights into the topic, the aim was to obtain in-depth and meaningful data about nurses' experiences with CR. To achieve this, one FG was conducted per hospital at each measurement. Consequently, it is uncertain whether data saturation was reached. No power analysis was conducted for the questionnaires, since the aim was to collect as many responses as possible through convenience sampling.

4.7 Data analysis

Analysis focus group interviews

Data of the FGs were analysed with directed content analysis. A strength of this kind of qualitative content analysis is that data are coded, divided, analysed, and identified systematically (Hsieh & Shannon, 2005). Data were structured directly into fixed preliminary codes, differentiating between quotations that referred to a facilitating, hindering or neutral quotation and assigned to fixed referring themes: mobility, nutrition, comfort rounding specific, or general aspects. An open coding was then adjusted to provide more information about the quote. Subsequently, codes were assigned to factors that influenced the implementation of an innovation: individual, social, organisational, and societal factors (Wensing et al., 2013b). These predetermined factors served as a framework to structure the

results and offered the possibility to compare findings between the eight FGs and different studies (Assarroudi et al., 2018). Data were stored and analysed with the computer-assisted qualitative data analysis software program ATLAS.ti (ATLAS.ti Scientific Software Development GmbH, Berlin, Germany, version 23.0.7.0) and Excel (Microsoft Corporation, 2024, Microsoft 365, version 2402).

Analysis of the questionnaire

Data of the questionnaire were processed in SPSS (IBM SPSS Statistics, version 29.0.0.0) and data were analysed with descriptive statistics.

4.8 Ethical considerations

This study was cleared for approval by the ethics committees in both centres (Hospital 1 METC 2021-2928, Hospital 2 METC 2021-13334) because it was assessed as not a subject to the Medical Research Involving Human Subjects Act. The study was carried out in accordance with the Declaration of Helsinki (World Medical Association, 2018) and the Netherlands' General Data Protection Regulation. Following local data management rules, study data were securely stored physically or digitally for ten years, accessible to four researchers (FP, YvE, GH, and FB).

Review Copy

4.9 Rigor and reflexivity

FGs were first coded by FB and FP (T0 and T2) and the bachelor's or master's students (T1 and T3). To increase the study's reliability, all focus group interviews were double coded independently from the first coding: FB and FP (T1 and T3), HvN and YvE (T0), and CR and YvE (T2). Initial coding and assignment to the four factors were discussed, and consensus was reached among the researchers (CR, FB, FP, YvE) after which a consensus document was drafted.

5. Findings

Acceptability and practicality of CR

In total, 32 instances of participation in focus group interviews were recorded, but some nurses participated more than once. Due to the use of convenience sampling, some overlap occurred, and the total number of unique nurse participants was 26. The FGs lasted between 34 and 70 minutes, with 3 to 5 nurses participating in each interview. Most participating nurses were female (See Appendix D, Table 1). Results of FG T0 were described separate from FG T1–T3 as these results illustrated ‘usual care’ before the introduction of CR. FG T1–T3 demonstrated results over time and were described integrative and per factor. When results were specific to one of the hospitals or one of the FGs, this was made explicit in the text or citation. Otherwise, it was applicable to both hospitals or all FGs. Furthermore, experiences were acquired by a questionnaire completed by nurses of both wards at T3 (see Appendix D, Table 2). At Hospital 1, 21 nurses (60%) and at Hospital 2, 22 nurses (67%) responded. Most respondents (76%) of Hospital 1 and all respondents (100%) of Hospital 2 were aware of CR and answered therefore question 8 till 11 of the questionnaire. Results of the FGs and questionnaire were presented separately; the FGs first and the questionnaire thereafter.

Review Copy

Focus group interviews

Baseline measurement: focus group interview T0

Before CR was introduced, nurses had positive expectations of structural attention to nutritional and mobility care because it could promote patient recovery and independence. CR was expected to be acceptable because nutritional and mobility care were already integrated into the day’s structure. However, time investment was an expected barrier to perform CR according to nurses of Hospital 2. These nurses indicated that CR has to be performed on top of the other work and could be skipped:

> *I think we already do it partly, but we don't schedule specific moments for it. I think we are always somewhat involved with the patient's eating and drinking and organising it, but we don't really involve the patient in it throughout the day. I think we could gain a lot from that, but as for time planning, we'll have to see.* (Respondent 2, Hospital 2, FG T0).

Acceptability and practicality of CR

Individual characteristics of nurses (e.g., knowledge, experience, priority-setting) influenced the structure, amount, and manner in which nurses paid attention to their patients' nutritional and mobility care, and this varied between nurses. Nurses' knowledge and skills were seen as supportive and important to facilitate personalised nutritional and mobility care, and several patient characteristics (e.g., motivation) were mentioned as hindering or facilitating nutritional and mobility care.

Focus group T1–T3: Individual factor

Nurses' attitude

During the implementation period, nurses stated that they already paid attention to their patients' nutritional intake and mobility in daily routines by observing, guiding, informing, advising, setting goals, motivating, and encouraging. CR did not appear to have any impact on these practices. However, CR may have influenced nurses at Hospital 2, as they furthermore mentioned discussing patients' needs, alternatives, and nurses' expectations. Additionally, they made appointments with patients and involved relatives when necessary. Structure, amount, and manner in which nurses paid attention to their patients' nutritional and mobility care still differed between individual nurses during the implementation period. These differences could be explained by individual characteristics of nurses such as attitude, age, skills, knowledge, experience, awareness, and the time the nurse felt was available to work on it.

> *… the older nurses, yes, they have been in the profession for a long time, and they know that. But the younger ones should be more involved in it as well.* (Respondent 4, Hospital 1, FG T3).

Nurses mentioned it was essential to match and discuss the patients' wishes and needs with what the nurses considered important for the patient because some patients were not easily encouraged or motivated to mobilise specifically. Nurses rated these cases as challenging.

Knowledge and skills

Review Copy

Acceptability and practicality of CR

During the implementation period, nurses reported some of their knowledge as insufficient (e.g. how to deal with unmotivated patients), and they noticed it could be refreshed or extended. Nurses of Hospital 2 mentioned the importance of knowledge and skills to deliver personalised nutritional and mobility care. Despite some insufficient knowledge, nurses of Hospital 2 indicated that personalised nutritional and mobility care was provided increasingly, and on a daily basis, during and at the end of the implementation period.

Acceptability and practicality in the individual factor

Findings revealed mixed opinions regarding nurses' evaluation of the acceptability and practicality of CR both during and at the end of the implementation period. During the implementation period, nurses mentioned a positive influence of CR on patients' recovery, and CR was seen as an addition to usual care. Nurses of Hospital 2 indicated that CR served as a reminder and heightened awareness to deliver nutritional and mobility care:

> *I think awareness is already a very good step… we do not have to, we cannot always report everything, but at least it is in our minds that we are working on it and that we have highlighted it at least once during the shift.* (Respondent 10, Hospital 2, FG T2)

However, despite the introduction of CR, most nurses still wished more awareness and intentional attention to nutritional and mobility care during the implementation period. At the end of the implementation period, nurses of Hospital 2 assessed CR as acceptable and practical because CR was part of usual care. These nurses considered CR valuable for new colleagues specifically because CR could provide structure and raise awareness. In contrast, nurses evaluated that CR was not completely performed by all nurses during and at the end of the implementation period. At the end of the implementation period, nurses rated CR as unimportant, experienced no differences between the period without and with CR, and did not perform CR with awareness because nutritional and mobility care were integrated in usual care already. According to these nurses, CR did not noticeably change or add value to usual care, leading to an unclear evaluation of the acceptability and practicality of CR in this individual factor. Respondent 5 of Hospital 1 commented, 'I find it strange that a structure needs to be imposed on daily tasks' (FG T3). Another nurse stated:

Review Copy

> *… I actually don't notice a big difference… I feel like it's already part of a kind of daily routine. You don't really notice the difference between with or without comfort rounding… I'm not performing it with awareness, thinking, oh now I'm going to do a comfort rounding, or now I'm going to say something, for example…* (Respondent 16, Hospital 2, FG T3)

Focus group T1–T3: Social factor

Patient characteristics

Patient characteristics either hindered or facilitated nutritional and mobility care, and CR did not seem to have had any influence according to the nurses. Individual characteristics of patients included tiredness, fear, memory, patients' behaviour (e.g. patients' wait-and-see attitude or imperviousness to stimulus), isolation precautions, and surgery-related factors—such as dietary restrictions and presence of tubes and lines— limited patients' willingness, possibilities, or permissions for nutritional and mobility care. Also, opposite beliefs, wishes, and needs between nurses and patients could result in struggles. Furthermore, talking too much about nutrition or mobility could actually be counterproductive:

> *…and I sometimes wonder about nutrition. Sometimes people say, 'You talk about nutrition all the time, but I am really not hungry'… that it kind of works against it, so to speak, and… that patients start to dig their heels in a bit more, that feeling.* (Respondent 7, Hospital 2, FG T2)

In contrast, patients' preoperative fitness, motivation, and positive experiences were facilitating mobility care.

Nurse-patient relationship

During the implementation period, nurses of Hospital 2 indicated that building a relationship between a nurse and a patient was essential to perform CR. CR resulted in sharing mutual

Review Copy

expectations with patients more often, and nurses obtaining better insight into patients' preferences and nutritional and mobility status. Furthermore, nurses gained this insight from more patients than before, and patients mobilised at an earlier moment.

Focus group T1–T3: Organisational factor

Workload, time pressure, and team culture

In the implementation period, nurses of both hospitals confirmed that workload and experienced time pressure were barriers for nutritional and mobility care and the performance of CR. This hindered the acceptability and practicality of CR. Specifically at Hospital 1, staff shortages and an increased workload and number of tasks were mentioned explanations. Furthermore, some nurses of Hospital 1 were not aware of CR during the implementation period due to perceived ineffective communication, and some nurses did not perform CR because they had not adequately informed themselves about CR. Nurses of Hospital 2 experienced CR as time consuming, as an extra task, and were searching for the best way to implement CR. Consequently, these nurses experienced that CR prevented them from doing other things and mentioned that CR could be skipped, forgotten, or not prioritised. Sometimes, the unpredictability of patient care made it difficult for nurses and patients to adhere to patients' individualised day structures or scheduled appointments regarding nutrition and mobility. This led to nurses who had to prioritise their work and had to make compromises that resulted in less patient participation, less information supply and support, less time to discuss with or respond to patients, and a modified way or duration of mobilisation. During and at the end of the implementation period, nurses of Hospital 1 explained that their team culture and nurse-colleagues' attitude were hindering the implementation of CR, and hindered its' acceptability, as it was the case with other implementations.

> *Because yes, it keeps being mentioned and everyone is nodding and agreeing. But in the end, the effect... it does not happen.* (Respondent 4, Hospital 1, FG T2).

Despite a hindering team culture, nurses of Hospital 1 mentioned that they discussed nutrition and mobility routinely during physician's visits at the end of the implementation

Review Copy

period, which suggested that CR may have had some positive impact on nutritional and mobility care.

Design and implementation

Only nurses of Hospital 2 mentioned that the design and implementation of CR influenced its acceptability and practicality. At first, some nurses evaluated the description of CR as 'too extensive':

> *When I see that form, I sometimes find it quite a lot to read at a glance what exactly is expected of me. So, I would sometimes really appreciate it if, for example, at 8:00 AM, I could see in three words what I need to discuss or at least something more organised, so to speak…* (Respondent 8, Hospital 2, FG T1)

Review Copy

Nurses found the original CR design too rigid due to fixed times, which was a barrier. Despite repeated focus on this topic, not all nurses understood the CR during implementation. After tailoring the design to allow flexible timing, Hospital 2 nurses accepted CR more and reported improved performance. By the end, nurses felt sufficiently informed but found reminders too frequent and intrusive, sometimes perceiving them as pedantic or questioning their performance. Thus, tailoring improved CR's acceptability and practicality, though implementation still did not fully meet nurses' preferences.

Reports, registration and handovers

Nurses' reports, electronic patient file entries, and handovers were seen as essential and supportive for nutritional and mobility care delivery. They helped ensure continuity by increasing awareness and insight into patients' status, and facilitated integration into daily routines and nurse communication—especially when problems or notable findings were present.

During implementation, nurses from both hospitals sometimes found reports and registrations to be incomplete, inconsistent, and insufficiently informative—hindering insight

into patients' nutritional intake and mobilisation, as well as continuity of care. At Hospital 1, this was mainly due to incompatibility between the electronic patient file and the nutrition registration system. At Hospital 2, a tailored smart phrase introduced after FG T1 helped raise awareness, improve reporting on nutritional and mobility status, support care evaluation, and highlight comfort rounding.

> *I think so too. Especially with nutrition, you see those calories listed, and then you think, I actually did not ask if it tasted good. So, it is just like with diuresis, but also with nutrition, you think, I need to go back and ask about that.* (Respondent 10, Hospital 2, FG T2)

At the end of the implementation period, nurses of Hospital 2 stated that there were no insufficiencies in nurses' reports anymore.

Review copy

Questionnaire

As demonstrated in Table 3, nurses of both hospitals provided attention to nutrition and mobility together with patients, performing CR was one of their priorities and respondents used the patient file when performing CR. Furthermore, nurses considered CR as of added value and helpful in providing structured attention to nutrition and mobility, and this appeared to apply slightly more to nurses at Hospital 1 than at Hospital 2. Respondents at Hospital 1 were neutral towards the statement that there was sufficient time to perform CR, while respondents at Hospital 2 indicated that the time was sufficient. Respondents of both hospitals indicated that CR were often not performed in the day and evening shift (Table 4). Nevertheless, respondents of Hospital 1 indicated that all components of CR were performed most of the time and respondents of Hospital 2 mentioned that the components of CR were performed most of the time till very often, except the evaluation of care with patients (Table 4).

[Insert Table 3. Question 8.1 to 8.7 of the questionnaire: the attitude of nurses towards comfort rounding*]

[Insert Table 4. Question 9 to 11.9 of the questionnaire: the execution of (several components of) comfort rounding by nurses*]

Acceptability and practicality of CR

Synthesis

Synthesis of the results demonstrates that nurses generally found CR to be acceptable and practical during the implementation period, as many of its elements aligned with existing care routines. It was regarded as a valuable addition that enhanced care delivery by raising awareness around nutrition and mobility, structuring attention to personalised care, and deepening interactions with patients and families. However, some nurses felt CR added little to their current practice. Its performance varied and was not consistently carried out across shifts, partly due to differences in nurses' knowledge, skills, and perceptions. Although most CR components were reportedly performed regularly, nurses acknowledged that CR was often not performed as intended during their shifts. This contradiction suggests that, while the content of CR was generally accepted and its components were performed, the concept of CR—with its fixed structure and design—was not always practical in everyday clinical practice. Nurses were divided on whether CR fit into their daily routines, with notable differences between hospitals. Team dynamics, rigid protocols, and frequent reminders sometimes reduced motivation and perceived usefulness. Additionally, high workload, time pressure, and unpredictable care demands were major barriers to perform CR as intended.

6. Discussion

The aim of this study was to evaluate nurses' perspectives on factors influencing the acceptability and practicality of CR, with a focus on personalised nutritional and mobility care. Data were collected through eight focus group interviews and were supported by data of a one-time questionnaire.

On the one hand, results demonstrated that nurses perceived CR as acceptable and practical because it could enhance and improve the delivery of personalised nutritional and mobility care, and most aspects of CR were already incorporated into and performed as part of usual care. On the other hand, low acceptability and practicality emerged, as nurses indicated that CR did not add value or change usual care. Nurses were divided on whether CR fit into their daily routines, and although most aspects of CR were performed, it was not carried out as intended. The results regarding CR's acceptability and practicality are therefore contradictory.

Acceptability and practicality of CR

Comparison with previous studies demonstrated similar contradicting results regarding CR's acceptability and practicality. CR could be seen as acceptable as nurses indicated that CR could support the delivery of comprehensive and consistent care to patients, could have added value to improve quality of care (Christiansen et al., 2018; Leamy et al., 2023; Ryan et al., 2019), and could inform and alert new colleagues in particular to perform fundamental patient care (Leamy et al., 2023; Ryan et al., 2019). However, previous research also demonstrated that roundings were not always applied to daily practice, were not consistently performed during allocated 'rounding' time, and were integrated into other patient care activities (Leamy et al., 2023), indicating a low acceptability and practicality of CR.

A possible explanation for the low acceptability of CR, could be found in the absence of a clear necessity to change usual personalised nutritional and mobility care into CR (Christiansen et al., 2018), as nurses in this study reported to perform most components of CR in usual care already and did not prefer a new structure to deliver personalised nutritional and mobility care. Another explanation for a low acceptability of CR could be found in the negative influence of CR on nurses' autonomy. Previous studies revealed that implementing CR could hinder nurses' professional autonomy if prescriptive and restrictive protocols prevent nurses from personalising their care or critically thinking (Christiansen et al., 2018; Ryan et al., 2019). Conversely, flexible and adaptive protocols regarding CR facilitate its implementation over time (Christiansen et al., 2018) and could positively influence the acceptability and practicality of CR, This was demonstrated at hospital 2, where the attitude to and performance of CR improved after tailoring to a more flexible design, although the questionnaire revealed that CR was still often not performed as intended by nurses.

Differences between hospitals—such as nurse-patient ratios, staff shortages, and perceived workload—may have contributed to the contrasting results as well. Prior research demonstrated that CR compliance and effectiveness were hindered, amongst others, by time constraints, workload, competing priorities, staffing issues, and difficulty integrating CR into daily routines (Christiansen et al., 2018; Leamy et al., 2023). Hospital 1 faced more challenges than Hospital 2 due to higher nurse-patient ratios, perceived lack of time, workload, and a restrictive team culture. Surprisingly, nurses at Hospital 1 still saw slightly

Review Copy

more added value in CR, though they noted few changes during the implementation period. Hospital 2, after tailoring CR, observed improved performance and increased patient participation. Despite these differences, no clear variation in CR's overall performance, acceptability, or practicality was found. This suggests that a positive attitude alone does not lead to behavioural change. Acceptability and practicality appear context-dependent and not generalisable. Future implementation should consider both positive expectations and barriers such as workload and team culture, while learning from the success of Hospital 2 in tailoring CR to practice.

In this feasibility study, evaluating the acceptability and practicality of CR is essential to determine its suitability for further investigation. Prior to initiating a full-scale study, it is important to carefully consider the positive and negative factors that influence CR's acceptability and practicality. A prerequisite for further testing is that nurses perceive a clear need to shift from conventional nutritional and mobility care practices to the CR approach. Additionally, it is crucial that the design of CR is tailored to align with current nursing practices and respects nurses' preferences for professional autonomy. This requires a careful co-design process involving a representative group of nurses from the participating ward. Finally, organisational support is required to enable the implementation and performance of CR, as factors such as workload, time constraints, and team culture have played a key role in the delivery of CR.

Review Copy

6.1 Strengths and limitations of the Work

Strengths of this study were found in the study design. CR were tested at two university medical centres for one year, and nurses were interviewed before, during, and at the end of the implementation period. The qualitative design fostered a broad and in-depth knowledge about nurses' experiences regarding CR, and the heterogeneity between the two hospitals may signify a difference in the role that CR could play in patient care. Furthermore, the design facilitated enough time for the researchers to inform, educate, and interview nurses, and resulted in enough time for nurses to experiment with CR and to discuss their experiences in the FGs or questionnaire. The quantitative results of the questionnaire supported the findings of the focus group interviews by capturing the perspectives of as many nurses as possible from both departments.

Acceptability and practicality of CR

Although a predominantly qualitative approach was deliberately chosen during the design phase to assess the acceptability and practicality of CR from nurses' perspectives, a more quantitative design could also have been selected, for example by using the Theoretical Framework of Acceptability (TFA) questionnaire. Using the TFA allows for standardised data collection across time points and provides an efficient means of gaining insight into potential challenges nurses may experience regarding the acceptability of CR (Sekhon et al., 2022). To our knowledge, this is the first study that investigated the acceptability and practicality of CR with attention specifically to nutritional and mobility care and patient participation. This study offered the opportunity to investigate if CR could contribute to the performance of fundamental nursing care (Kitson, 2018). Prior literature demonstrated similar results regarding the acceptability and practicality of CR, although the content of the roundings was not similar. Given the substantial overlap in findings, it seems that the specific content of CR may not be the main factor influencing its acceptability and practicality. Instead, this study highlighted that the structure and flexibility of rounding in usual care seem to matter more than the exact details discussed during rounds. Some limitations were found in the role of the researcher of Hospital 2, differences in educational levels between respondents of the two hospitals, and the challenge at both hospitals to recruit participants for the FG. The researcher from Hospital 2 (FB) worked as a clinical academic nurse, combining bedside nursing care with nursing science. She was present during the FGs at Hospital 2. This could have led to participant bias, where participants gave socially desirable answers, or confirmation bias, where the researcher interpreted results in favour of her hypothesis. To minimise bias and enhance methodological rigour, member checking, double coding, and involvement of independent researchers were implemented and were therefore strengths of this study. Another limitation was the varying educational levels of the nurses who completed the questionnaire. This led to less homogeneous settings and less clear generalisability of the results. Also, the challenge to recruit nurses to participate in the focus group interviews led to convenience sampling with a minimum of participants in each focus group interview. The heterogeneity and convenience sampling in this study might have biased the results.

Review Copy

6.2 Recommendations for Further Research

Acceptability and practicality of CR

A different approach to personalised nutritional and mobility care is needed, as nutritional and mobility care are vulnerable to being left undone. This study highlighted that a fixed structure is not desired, that nurses' performance of nutritional and mobility care varies and remains too dependent on the individual knowledge and skills of nurses. Future research should investigate effective approaches for the consistent delivery of high-quality, personalised nutritional and mobility care, without too fixed structures, allowing for nurses' autonomy, and regardless of which nurse is providing the care. A Delphi study involving multiple nurses from different hospitals could help address this question. Despite tasks and responsibilities being similar across educational levels, future research could explore whether differences in nurses' education influence attitudes towards personalised nutritional and mobility care delivery and comfort rounding, as well as whether these differences lead to varying needs for tailoring such care and which approaches are most effective for each group in achieving personalised care. Furthermore, nurse managers' perspectives were not examined in this study; however, it is essential that future research incorporates their views when developing and implementing structured approaches to personalised nutritional and mobility care. Their insights can drive organisational change and support the successful adoption of interventions. Moreover, the effects of personalised nutritional and mobility care on patients' nutritional or mobilisation status and patients' experienced participation are still unknown. Future research should investigate if personalised nutritional and mobility care and patient participation affect patient outcomes, such as nutritional and mobility status, satisfaction, or self-care. If a positive trend becomes visible, it serves as evidence and an extra incentive for nurses to develop and organise their care in a way that enables them to provide this personalised care as well.

6.3 Implications for policy and practice

Before implementing CR, it is important to consider several key points. First, determine if there is a need to change the usual care. Second, CR needs to be tailored to the specific setting where it is implemented to allow nurses flexibility in their shifts and to personalise their care to their patients' needs. Finally, the team must be prepared and ready to undertake such an implementation, ensuring that all nurses make a concerted effort towards achieving a successful implementation.

Review Copy

Acceptability and practicality of CR

7. Conclusion

CR is acceptable and practical in daily practice when flexibility in execution is allowed, because nurses value its importance and usefulness to improve attention for patients' nutritional and mobility care, and patient participation. Individual, social, and organisational factors included both facilitators and barriers to the acceptability and practicality of CR. When components of CR were already incorporated in usual care and performed by nurses, nurses were resistant to implementing a fixed schedule to perform this aspect of usual care which hindered CR's acceptability and practicality. Although nurses did not consider the CR concept crucial, the implementation period led to notable improvements in nutritional and mobility care, as well as patient participation, according to the nurses.

Review Copy

References

Assarroudi, A., Heshmati Nabavi, F., Armat, M. R., Ebadi, A., & Vaismoradi, M. (2018). Directed qualitative content analysis: the description and elaboration of its underpinning methods and data analysis process. *Journal of research in nursing*, *23*(1), 42-55.

Bowen, D. J., Kreuter, M., Spring, B., Cofta-Woerpel, L., Linnan, L., Weiner, D., Bakken, S., Kaplan, C. P., Squiers, L., Fabrizio, C., & Fernandez, M. (2009). How we design feasibility studies. *Am J Prev Med*, *36*(5), 452-457. https://doi.org/10.1016/j.amepre.2009.02.002

Christiansen, A., Coventry, L., Graham, R., Jacob, E., Twigg, D., & Whitehead, L. (2018). Intentional rounding in acute adult healthcare settings: Asystematic mixed-method review. *Journal of Clinical Nursing*, *27*(9-10), 1759-1792. https://doi.org/10.1111/jocn.14370

Correia, M. I., & Waitzberg, D. L. (2003). The impact of malnutrition on morbidity, mortality, length of hospital stay and costs evaluated through a multivariate model analysis. *Clin Nutr*, *22*(3), 235-239. https://doi.org/10.1016/s0261-5614(02)00215-7

Daniels, J. F. (2016). Purposeful and timely nursing rounds: a best practice implementation project. *JBI Evidence Synthesis*, *14*(1), 248-267.

de Almeida, E. P. M., de Almeida, J. P., Landoni, G., Galas, F., Fukushima, J. T., Fominskiy, E., de Brito, C. M. M., Cavichio, L. B. L., de Almeida, L. A. A., Ribeiro, U., Jr., Osawa, E. A., Diz, M. P., Cecatto, R. B., Battistella, L. R., & Hajjar, L. A. (2017). Early mobilization programme improves functional capacity after major abdominal cancer surgery: a randomized controlled trial. *Br J Anaesth*, *119*(5), 900-907. https://doi.org/10.1093/bja/aex250

Di Massimo, D. S., Catania, G., Crespi, A., Fontanella, A., Manfellotto, D., La Regina, M., De Carli, S., Rasero, L., Gatta, C., Pentella, G., Bordin, G., Croso, A., Bagnasco, A., Gussoni, G., Campani, D., Busca, E., Azzolina, D., Dal Molin, A., & Group, o. b. o. t. I. S. (2022). Intentional Rounding versus Standard of Care for Patients Hospitalised in Internal Medicine Wards: Results from a Cluster-Randomised Nation-Based Study. *Journal of Clinical Medicine*, *11*(14), 3976. https://doi.org/10.3390/jcm11143976

EPUAP, NPIAP, & PPPIA. (2019). Prevention and Treatment of Pressure Ulcers/Injuries: Clinical Practice Guideline. The international guideline. http://www.internationalguideline.com/static/pdfs/Quick_Reference_Guide-10Mar2019.pdf

Fazio, S., Stocking, J., Kuhn, B., Doroy, A., Blackmon, E., Young, H. M., & Adams, J. Y. (2020). How much do hospitalized adults move? A systematic review and meta-analysis. *Appl Nurs Res*, *51*, 151189. https://doi.org/10.1016/j.apnr.2019.151189

Feo, R., Conroy, T., Jangland, E., Muntlin Athlin, Å., Brovall, M., Parr, J., Blomberg, K., & Kitson, A. (2018). Towards a standardised definition for fundamental care: A modified Delphi study. *J Clin Nurs*, *27*(11-12), 2285-2299. https://doi.org/10.1111/jocn.14247

Feo, R., Conroy, T., Marshall, R. J., Rasmussen, P., Wiechula, R., & Kitson, A. L. (2017). Using holistic interpretive synthesis to create practice-relevant guidance for person-centred fundamental care delivered by nurses. *Nurs Inq*, *24*(2). https://doi.org/10.1111/nin.12152

Ferreyra, G., Long, Y., & Ranieri, V. M. (2009). Respiratory complications after major surgery. *Current Opinion in Critical Care*, *15*(4), 342-348. https://doi.org/10.1097/MCC.0b013e32832e0669

Griffiths, P., Recio-Saucedo, A., Dall'Ora, C., Briggs, J., Maruotti, A., Meredith, P., Smith, G. B., & Ball, J. (2018). The association between nurse staffing and omissions in nursing care: A systematic review. *J Adv Nurs*, *74*(7), 1474-1487. https://doi.org/10.1111/jan.13564

Grol, R., Bosch, M., & Wensing, M. (2013). Development and selection of strategies for improving patient care. In *Improving Patient Care* (pp. 165-184). https://doi.org/https://doi.org/10.1002/9781118525975.ch10

Harris, R., Sims, S., Leamy, M., Levenson, R., Davies, N., Brearley, S., Grant, R., Gourlay, S., Favato, G., & Ross, F. (2019). *Health Services and Delivery Research* (Intentional rounding in hospital

Review Copy

wards to improve regular interaction and engagement between nurses and patients: a realist evaluation, Issue. https://njl-admin.nihr.ac.uk/document/download/2030737

Hsieh, H.-F., & Shannon, S. (2005). Three Approaches to Qualitative Content Analysis. *Qualitative health research*, *15*, 1277-1288. https://doi.org/10.1177/1049732305276687

Jakobson, T., Karjagin, J., Vipp, L., Padar, M., Parik, A. H., Starkopf, L., Kern, H., Tammik, O., & Starkopf, J. (2014). Postoperative complications and mortality after major gastrointestinal surgery. *Medicina (Kaunas)*, *50*(2), 111-117. https://doi.org/10.1016/j.medici.2014.06.002

Kalisch, B. J., Lee, S., & Dabney, B. W. (2014). Outcomes of inpatient mobilization: a literature review. *J Clin Nurs*, *23*(11-12), 1486-1501. https://doi.org/10.1111/jocn.12315

Khuri, S. F., Henderson, W. G., DePalma, R. G., Mosca, C., Healey, N. A., Kumbhani, D. J., & Program, t. P. i. t. V. N. S. Q. I. (2005). Determinants of Long-Term Survival After Major Surgery and the Adverse Effect of Postoperative Complications. *Annals of Surgery*, *242*(3), 326-343. https://doi.org/10.1097/01.sla.0000179621.33268.83

Kitson, A. L. (2018). The Fundamentals of Care Framework as a Point-of-Care Nursing Theory. *Nurs Res*, *67*(2), 99-107. https://doi.org/10.1097/nnr.0000000000000271

Koenders, N., Marcellis, L., Nijhuis-van der Sanden, M. W., Satink, T., & Hoogeboom, T. J. (2021). Multifaceted interventions are required to improve physical activity behaviour in hospital care: a meta-ethnographic synthesis of qualitative research. *J Physiother*, *67*(2), 115-123. https://doi.org/10.1016/j.jphys.2021.02.012

Koenders, N., van Oorsouw, R., Seeger, J. P. H., Nijhuis – van der Sanden, M. W. G., van de Glind, I., & Hoogeboom, T. J. (2020). "I'm not going to walk, just for the sake of walking…": a qualitative, phenomenological study on physical activity during hospital stay. *Disability and Rehabilitation*, *42*(1), 78-85. https://doi.org/10.1080/09638288.2018.1492636

Krueger, R. A. (2014). *Focus groups: A practical guide for applied research*. Sage publications. https://books.google.nl/books?hl=nl&lr=&id=8wASBAAAQBAJ&oi=fnd&pg=PP1&ots=XfjNEtdOtR&sig=sUKuISv-2KOSK8409pDtfTxcZ9s&redir_esc=y#v=onepage&q&f=false

Kruizenga, H., van Keeken, S., Weijs, P., Bastiaanse, L., Beijer, S., Huisman-de Waal, G., Jager-Wittenaar, H., Jonkers-Schuitema, C., Klos, M., Remijnse-Meester, W., Witteman, B., & Thijs, A. (2016). Undernutrition screening survey in 564,063 patients: patients with a positive undernutrition screening score stay in hospital 1.4 d longer. *Am J Clin Nutr*, *103*(4), 1026-1032. https://doi.org/10.3945/ajcn.115.126615

Larsson, I. E., Sahlsten, M. J., Sjöström, B., Lindencrona, C. S., & Plos, K. A. (2007). Patient participation in nursing care from a patient perspective: a Grounded Theory study. *Scand J Caring Sci*, *21*(3), 313-320. https://doi.org/10.1111/j.1471-6712.2007.00471.x

Leamy, M., Sims, S., Levenson, R., Davies, N., Brearley, S., Gourlay, S., Favato, G., Ross, F., & Harris, R. (2023). Intentional rounding: a realist evaluation using case studies in acute and care of older people hospital wards. *BMC Health Serv Res*, *23*(1), 1341. https://doi.org/10.1186/s12913-023-10358-1

Ljungqvist, O., & Hubner, M. (2018). Enhanced recovery after surgery—ERAS—principles, practice and feasibility in the elderly. *Aging Clinical and Experimental Research*, *30*(3), 249-252. https://doi.org/10.1007/s40520-018-0905-1

Mitchell, P. H. (2008). Defining Patient Safety and Quality Care. In R. G. Hughes (Ed.), *Patient Safety and Quality: An Evidence-Based Handbook for Nurses.* (pp. 1-4). Agency for Healthcare Research and Quality. https://www.ncbi.nlm.nih.gov/books/NBK2681/

Morgan, D. L. (1996). *Focus groups as qualitative research* (Vol. 16). Sage publications. https://books.google.nl/books?hl=nl&lr=&id=LxF5CgAAQBAJ&oi=fnd&pg=PT6&ots=3uTnxP1WM5&sig=cNUgIFdFcy80s1E1EwnQnFMYSEo&redir_esc=y#v=onepage&q&f=false

Nydahl, P., Jeitziner, M.-M., Vater, V., Sivarajah, S., Howroyd, F., McWilliams, D., & Osterbrink, J. (2023). Early mobilisation for prevention and treatment of delirium in critically ill patients: Systematic review and meta-analysis. *Intensive and Critical Care Nursing*, *74*, 103334. https://doi.org/https://doi.org/10.1016/j.iccn.2022.103334

Review Copy

Rios, T. C., de Oliveira, L. P. M., da Costa, M. L. V., da Silva Baqueiro Boulhosa, R. S., Roriz, A. K. C., Ramos, L. B., & Bueno, A. A. (2021). A poorer nutritional status impacts quality of life in a sample population of elderly cancer patients. *Health Qual Life Outcomes*, *19*(1), 90. https://doi.org/10.1186/s12955-021-01735-7

Ryan, L., Jackson, D., Woods, C., & Usher, K. (2019). Intentional rounding - An integrative literature review. *J Adv Nurs*, *75*(6), 1151-1161. https://doi.org/10.1111/jan.13897

Sahlsten, M. J., Larsson, I. E., Sjöström, B., Lindencrona, C. S., & Plos, K. A. (2007). Patient participation in nursing care: towards a concept clarification from a nurse perspective. *J Clin Nurs*, *16*(4), 630-637. https://doi.org/10.1111/j.1365-2702.2006.01660.x

Sekhon, M., Cartwright, M., & Francis, J. J. (2022). Development of a theory-informed questionnaire to assess the acceptability of healthcare interventions. *BMC Health Serv Res*, *22*(1), 279. https://doi.org/10.1186/s12913-022-07577-3

Sharma, A., Minh Duc, N. T., Luu Lam Thang, T., Nam, N. H., Ng, S. J., Abbas, K. S., Huy, N. T., Marušić, A., Paul, C. L., Kwok, J., Karbwang, J., de Waure, C., Drummond, F. J., Kizawa, Y., Taal, E., Vermeulen, J., Lee, G. H. M., Gyedu, A., To, K. G.,…Karamouzian, M. (2021). A Consensus-Based Checklist for Reporting of Survey Studies (CROSS). *J Gen Intern Med*, *36*(10), 3179-3187. https://doi.org/10.1007/s11606-021-06737-1

Sims, S., Leamy, M., Davies, N., Schnitzler, K., Levenson, R., Mayer, F., Grant, R., Brearley, S., Gourlay, S., Ross, F., & Harris, R. (2018). Realist synthesis of intentional rounding in hospital wards: exploring the evidence of what works, for whom, in what circumstances and why. *BMJ Qual Saf*, *27*(9), 743-757. https://doi.org/10.1136/bmjqs-2017-006757

Ten Cate, D., Mellema, M., Ettema, R. G. A., Schuurmans, M. J., & Schoonhoven, L. (2021). Older Adults' and Their Informal Caregivers' Experiences and Needs regarding Nutritional Care Provided in the Periods before, during and after Hospitalization: A Qualitative Study. *J Nutr Gerontol Geriatr*, *40*(2-3), 80-107. https://doi.org/10.1080/21551197.2021.1906822

Tong, A., Sainsbury, P., & Craig, J. (2007). Consolidated criteria for reporting qualitative research (COREQ): a 32-item checklist for interviews and focus groups. *Int J Qual Health Care*, *19*(6), 349-357. https://doi.org/10.1093/intqhc/mzm042

van Belle, E., Giesen, J., Conroy, T., van Mierlo, M., Vermeulen, H., Huisman-de Waal, G., & Heinen, M. (2020). Exploring person-centred fundamental nursing care in hospital wards: A multi-site ethnography. *J Clin Nurs*, *29*(11-12), 1933-1944. https://doi.org/10.1111/jocn.15024

van den Berg, G., de van der Schueren, M., Vermeulen, H., & Huisman-de Waal, G. (2023). Opportunities for Patient Participation in Perioperative Malnutrition Care: A Qualitative Study. *SAGE Open Nurs*, *9*, 23779608231193743. https://doi.org/10.1177/23779608231193743

Van Den Berg, G., Vermeulen, H., Conroy, T., Van Noort, H., De Van Der Schueren, M., & Huisman-de Waal, G. (2023). Factors influencing the delivery of nutritional care by nurses for hospitalised medical patients with malnutrition; a qualitative study. *J Clin Nurs*, *32*(15-16), 5147-5159. https://doi.org/10.1111/jocn.16614

van den Berg, G. H., Huisman-de Waal, G. G. J., Vermeulen, H., & de van der Schueren, M. A. E. (2021). Effects of nursing nutrition interventions on outcomes in malnourished hospital inpatients and nursing home residents: A systematic review. *Int J Nurs Stud*, *117*, 103888. https://doi.org/10.1016/j.ijnurstu.2021.103888

van der Leeden, M., Huijsmans, R., Geleijn, E., de Lange-de Klerk, E. S., Dekker, J., Bonjer, H. J., & van der Peet, D. L. (2016). Early enforced mobilisation following surgery for gastrointestinal cancer: feasibility and outcomes. *Physiotherapy*, *102*(1), 103-110. https://doi.org/10.1016/j.physio.2015.03.3722

Van Den Berg, G., Vermeulen, H., Conroy, T., Van Noort, H., De Van Der Schueren, M., & Huisman-de Waal, G. (2023). Factors influencing the delivery of nutritional care by nurses for hospitalised medical patients with malnutrition; a qualitative study. *Journal of Clinical Nursing*, *32*(15-16), 5147-5159. https://doi.org/https://doi.org/10.1111/jocn.16614

Veličković, J., Feng, C., Palibrk, I., Veličković, D., Jovanović, B., & Bumbaširević, V. (2020). The Assessment of Complications After Major Abdominal Surgery: A Comparison of Two Scales. *J Surg Res*, *247*, 397-405. https://doi.org/10.1016/j.jss.2019.10.003

Visioni, A., Shah, R., Gabriel, E., Attwood, K., Kukar, M., & Nurkin, S. (2018). Enhanced Recovery After Surgery for Noncolorectal Surgery?: A Systematic Review and Meta-analysis of Major Abdominal Surgery. *Ann Surg*, *267*(1), 57-65. https://doi.org/10.1097/sla.0000000000002267

Wensing, M., Bosch, M., & Grol, R. (2013a). Determinants of change. In *Improving patient care. The implementation of change in health care* (2 ed., pp. 139 - 150). John Wiley & Sons, Ltd. https://books.google.nl/books?id=oEEzUjFbDM8C&lpg=PT9&ots=6ZgkQjkLWO&dq=Improving%20Patient%20Care%3A%20The%20Implementation%20of%20Change%20in%20Health%20Care%2C%20Third%20Edition%20Chapter%203%20Effective%20Implementation%20of%20Change%20in%20Healthcare&lr&hl=nl&pg=PT30#v=onepage&q&f=false

Wensing, M., Bosch, M., & Grol, R. (2013b). Determinants of change. In *Improving Patient Care* (pp. 137-150). https://doi.org/https://doi.org/10.1002/9781118525975.ch8

World Medical Association. (2018). *WMA declaration of Helsinki - Ethical principles for medical research involving human subjects*. https://www.wma.net/policies-post/wma-declaration-of-helsinki-ethical-principles-for-medical-research-involving-human-subjects/

Review Copy

Table 1. Timeline of data collection and activities regarding the study

**Study stages***	**T0**	**Implementation**	**T1**	**T2**	**T3**
**Period**	September 2021–February 2022	March 2022	April–May 2022	June–December 2022	January–May 2023
**Measurement**	Baseline measurement		Intermediate measurement	Intermediate measurement	Follow-up measurement
**Data collection**	Focus group interview T0 (February ’22)		Focus group interview T1 (April ’22)	Focus group interview T2 (December ’22)	Focus group interview T3 (April ’23) Questionnaire (May ’23)
**Implementation phase (1)**	Orientation, Insight	Insight, Acceptance, Change	Change	Change	Sustainability
**Comfort rounding (CR)**	Preparing and constructing CR	Tailoring CR, Introduction of CR	Tailoring CR	Tailoring CR	Evaluation of CR
**Activities**	Informing colleagues in different ways, collecting nurses’ opinions about CR	Giving feedback from the focus group interview T0 to nurses, discussing results, constructing CR with input and feedback of colleagues	Data collection to collect experiences, tailoring CR.	Data collection to collect experiences, tailoring CR	Data collection to evaluate CR

*The study comprised four stages: T0 (baseline measurement), T1 and T2 (intermediate measurements), and T3 (follow-up measurement)

Review Copy

References:

1. Grol R, Bosch M, Wensing M. Development and selection of strategies for improving patient care. Improving Patient Care2013. p. 165-84.

Table 2. Development and tailoring of comfort rounding (CR)

Study stage*	Hospital 1	Hospital 2
**After T0**	• a schedule and content for CR was drafted at the request of nurses<br>• the schedule was visibly placed (e.g. computers, nurses' stations)	• a schedule and content for CR was drafted in co-creation with nurse colleagues<br>• the schedule was visibly placed (e.g. computers, nurses' stations)
**After T1**	• Patients received the same schedule as nurses at the wall of their room<br>• Nurses received a teaching moment<br>• Information about CR was placed on digital screens in the hallway	• A smart phrase was introduced in the nurses' reports of the patient file to remind nurses to report on their patients' nutritional intake and mobility<br>• Nurses no longer needed to adhere to the fixed schedule as they indicated that they needed more freedom to plan attention to nutrition and mobility themselves<br>• Reminders to perform CR were arranged (e.g. quiz, newsletter)
**After T2**	• Nurses received reminders to perform CR (e.g. discussion moment, emphasis on CR during day start and day end, tailored information on screens in hallways).	• The dietician provided teaching moments on nutrition on nurses' request<br>• The smart phrase in nurses' reports was changed because nurses indicated that the 'old' smart phrase resulted in double reporting about nutrition and mobility<br>• Some nurses indicated they wanted an extra reminder to perform CR in the patient file, some did not. To investigate what the majority wanted, a poll was conducted through the mail, which resulted in the rejection of an additional reminder in the patient file.

*T0 is the 'baseline measurement', T1 and T2 are 'intermediate measurements'

Review Copy

Table 3. Items 8.1 to 8.7 of the questionnaire: the attitude of nurses towards comfort rounding*

Items	Hospital 1 median (IQR)** n=16	Hospital 2 median (IQR)** n=22	Hospital 1 and 2 median (IQR)** n=38
8.1 Comfort rounding helps me in providing structured attention to nutrition and mobility	4 (3–4) n=16	3 (2–4) n=22	4 (3–4) n=38
8.2 Together with the patient, I provide attention to nutrition and mobility	4 (4–4) n=16	4 (4–5) n=22	4 (4–5) n=38
8.3 The current implementation of comfort rounding match daily practice	3 (3–4) n=16	3 (3–4) n=22	3 (3–4) n=38
8.4 Comfort rounding are not of added value for me	2 (1,5–2,5) Reversed score: 4 (3,5–4,5) n=16	3 (2–4) Reversed score: 3 (2–4) n=21	2 (2–3) Reversed score: 4 (2,5–4) n=37
8.5 I have sufficient time to perform comfort rounding	3 (2–3) n=16	4 (3–4) n=21	3 (3–4) n=37
8.6 I use the patient file (EPIC/SAP) when I perform comfort rounding	4 (3–4) n=15	4 (3–4) n=22	4 (3–4) n=37
8.7 Performing comfort rounding is not one of my priorities during my shift	2 (2–3) Reversed score: 4 (3–4) n=16	2 (2–3) Reversed score: 4 (3–4) n=21	2 (2–3) Reversed score: 4 (2–4) n=37

* Answers on a five-point Likert scale: 1 'strongly disagree', 2 'disagree', 3 'neutral', 4 'agree', 5 'strongly agree'

**Results shown in median Likert scores and interquartile range (IQR), measured according to Tukey's Hinges

Review Copy

Table 4. Items 9 to 11.9 of the questionnaire: the execution of (several components of) comfort rounding by nurses*

**Items**	**Hospital 1** median (IQR)**	**Hospital 2** median (IQR)**	**Hospital 1 and 2** median (IQR)**
9. Think of the day shifts of the last four weeks, how often do you perform comfort rounding?	2 (2–2,5) n=16	2 (2–2) n=21	2 (2–2) n=37
10. Think of the evening shifts of the last four weeks, how often do you perform comfort rounding?	3 (2–3) n=14	2 (2–2) n=20	2 (2–3) n=34
11.1 I discuss the nutritional and mobility status with patients	4 (4–5) n=16	5 (4–5) n=21	5 (4–5) n=37
11.2 I inform patients of the importance of nutrition and mobility	4 (4–5) n=16	5 (4–5) n=21	4 (4–5) n=37
11.3 I support patients in the execution of nutritional and mobility interventions	4 (4–4) n=16	5 (4–5) n=21	4 (4–5) n=37
11.4 I stimulate or motivate patients to eat/drink and move	4 (4–5) n=16	5 (4–5) n=21	4 (4–5) n=37
11.5 I give advice and discuss possibilities/alternatives about nutrition and mobility with patients	4 (4–4,5) n=16	4 (4–5) n=21	4 (4–5) n=37
11.6 I discuss patients' wishes, expectations and needs for nutrition and mobility	4 (3–4,5) n=16	4 (4–4) n=22	4 (4–4) n=38
11.7 I make agreements with patients on nutritional intake and mobility	4 (4–4) n=16	4 (4–5) n=22	4 (4–5) n=38

Review Copy

11.8 I execute the agreements made with patients about nutrition and mobility	4 (4–4,5)<br><br>n=16	4 (4–5)<br><br>n=22	4 (4–5)<br><br>n=38
11.9 I evaluate care related to nutrition and mobility with the patient	4 (4–5)<br><br>n=16	3,5 (3–4)<br><br>n=22	4 (3–4)<br><br>n=38

*Answers on a five-point Likert scale: 1 'never', 2 'most of the time not', 3 'as often as not', 4 'most of the time', 5 'very often/always'

**IQR = interquartile range, measured according to Tukey's Hinges

Review Copy

Appendix B. Interview protocol focus group interviews T0 and T1-T3*

Focus group interview protocol T0

Background

This interview protocol has been developed for focus group interviews with nurses to determine whether comfort rounding with attention to mobility and nutritional intake is feasible/applicable in practice and how nurses experience this.

Purpose of the interview

The purpose of the focus group interviews during T0 is:

- To gain insight into the inhibiting and promoting factors of care regarding nutrition and exercise.
- To coordinate the design of comfort rounding (applicability and feasibility).
- And whether, and if so, what support materials are needed to optimize care regarding nutrition and exercise.

Review Copy

Central themes

The focus group interview has 3 central themes:

- Facilitating and hindering factors regarding care for nutrition and mobility.
- Feasibility of comfort rounding.
- Required support materials for optimising care.

The themes are reflected in central questions. These questions cover all aspects of the used implementation model (1).

Where the discussion does not get going well or where certain topics are insufficiently covered, additional questions have been prepared.

**Semi-structured focus groep interview**

3.1 Introduction + opening question	5-10 min
Care for mobilisation and nutrition is essential for the recovery of the surgical patient. Separately and combined, mobility and adequate nutritional intake can lead to, among other things, preservation of function, improved self-care, shortened hospital stay, fewer complications, and have a positive impact on confidence in recovery and psychological well-being.<br><br>For this study, it is important to know how you, as a nurse, experience the care around movement and nutrition.<br><br>"Can you tell us how you experience providing this care?"	
**3.1 Interview sub topics**	**40-50 min**
**A.1. Attention**<br>"You have just shared your experiences of providing care in relation to mobility and nutrition (brief summary). Could you also tell us how you pay attention to mobility and nutrition?"	
Additional questions	
- What activities related to patients' movement and nutrition take place during a day?<br>- When do you apply these activities? (at fixed times?)<br>- How do you do this? (encouraging self-care? providing information?)<br>- Where do you document this?	
**A.2. Encouraging/motivating**<br>You have just indicated how you pay attention to mobility and nutrition in your patient (brief summary). For some patients, mobilising and ensuring a good intake of nutrition happens naturally, while for others, it is more challenging. This can have multiple causes, including a lack of motivation from the patient.	

Review Copy

"How do you deal with this?" ("Do you pay attention to encouraging and motivating your patient?" And if so, "how?")
Additional questions
- Do you encourage patients to move/eat? How do you do this?<br>- How do you support the patients?
**A.3. Insight into Mobility/Nutrition Behaviour (Participation)**<br>Insight into mobility and nutrition behaviour gives you, as a nurse, an indication of how your patient's recovery is progressing. "How do you gain insight into the mobility and nutrition behaviour of your patients?"
**A.4. Patient participation**<br>We have discussed attention, patient motivation, and insight into the mobility and nutrition behaviour of your patients.<br><br>"Do you take your patient's preferences into account when making arrangements about mobility and nutrition? And how do you do that?"
Additional questions
- Do you involve patients in the care related to mobility and nutrition? How do you do this?<br>- Do you tailor the care to the personal needs of the patients? How do you do that?
**A.4.a. Informing/education**<br>From patient participation, transitioning to: "How do you inform patients about mobility and nutrition?"
Additional questions

Review Copy

- What additional materials do you use for this?
**A.5. Knowledge/skills**<br>You always bring your knowledge and expertise with you when you talk to your patient, to inform them but also to motivate/encourage them. “How is this knowledge?” “And what is needed?”
Additional questions
- To what extent do you feel you have knowledge about the benefits of mobility and nutrition?<br>- To what extent do you know how to discuss the benefits of nutritional intake/movement with patients?<br>- What knowledge do you need to provide your patient with reliable information?<br>- Is it clear to you whom you can turn to for questions regarding patients' mobility and nutrition?
**B.1. Team attention**<br>We have just spoken very individually about how you, as a nurse, pay attention to mobility and nutrition. In caring for your patient, you are part of a multidisciplinary team. “Therefore, I would like to know how attention is paid to mobility and nutrition within that multidisciplinary team?”
Additional questions
- What agreements are in place regarding this?<br>- How is continuity of care ensured?<br>- What is your expectation if there is more consistent attention to movement and nutrition?<br>- If we are going to approach this more systematically, what do you need?
**B.2. Rol perception team**

Review Copy

Brief summary of the team's attention. “How do you see your role as a nurse regarding care for nutrition, movement, and patient participation in relation to the medical and paramedical staff?”
Additional questions
- Is care coordinated among the team? How is this done?
**B.3. Influence (role) family/relatives**<br>In caring for your patient, you also deal with concerns from the family/relatives. “Do family/relatives have an influence on the mobility and nutrition behaviour of patients (positive/negative)? “How”
” Do you, as a nurse, have an influence on this? How? Do you ever use the influence of family/relatives to promote the movement and nutrition behaviour of patients? How?
**C.1. Organisation of care**<br>We have already discussed how attention is paid to mobility and nutrition as a multidisciplinary team. “How is this care organised in your department?”
Additional questions
- How are tasks divided among the different disciplines? (multidisciplinary collaboration)<br>- Through which channels do the involved parties communicate?<br>- How does decision-making take place?<br>- How are the care processes related to mobility and nutrition organised?
**C.2. Environmental factors**<br>Research has shown that environmental factors also play an important role in mobility and nutrition. “How do you see this?”
**D.1. Facilitating factors**<br>We have discussed a lot so far, and it's great to get this input from you. Many things have been discussed that are going well, but also things that you think could be

Review Copy

improved or done differently. Therefore, I would first like to give a summary of what is going well in each area and hear if I have understood correctly and if you have any additions. After that, I would also like to do the same for the things that can be improved or that you would prefer to see done differently. In summary, the following has been discussed and you have indicated that these are going well:<br>- Individual factors (attention, motivation, insight into mobility and nutrition behaviour, patient participation, knowledge/skills, informing/education)<br>- Social factors (team attention, team role perception, influence of family/relatives)<br>- Organisational factors (organisation of care, environmental factors)<br>- Societal factors
Additional questions
- What do you think is going well?<br>- And why do you think that is?
**E.1. Hindering factors**<br><br>As mentioned, I would like to do the same with the things that you think could be improved or done differently. I will give a summary of each area to hear if I have understood correctly and if you have any additions.<br><br>- Individual factors (attention, motivation, insight into movement and nutrition behaviour, patient participation, knowledge/skills, informing/education)<br>- Social factors (team attention, team role perception, influence of family/relatives)<br>- Organisational factors (organisation of care, environmental factors)<br>- Societal factors
Additional questions
- What do you think is going well?

Review Copy

- And why do you think that is?

F.1. Expectation of comfort rounding

You have provided a lot of input about the current care regarding movement and nutrition. This has given me insight into what is going well and what can be improved. It is clear that you can (and want to) make a significant contribution to optimal care for movement and nutrition. Studies show that a possible beneficial intervention is the implementation of comfort rounding. The aim of this study is to determine whether comfort rounding with attention to mobility and nutritional intake is feasible/applicable in practice.

“Are you familiar with comfort rounding?”

In comfort rounding, nurses visit patients at fixed times and have structured attention for predetermined care aspects. Research shows that comfort rounding has a positive effect on patient satisfaction and essential care aspects, such as forming a professional relationship with patients and reducing fall incidents and pressure ulcers. More research is needed to demonstrate whether comfort rounding is effective and how to implement it. We would like to further specify this together with you.

Hearing this, “What is your expectation of applying comfort rounding?”

Additional questions

- What benefits do you see?
- What disadvantages do you see?

F.2. Designing comfort rounding

“How can we best implement comfort rounding in your department?”

Additional questions

- What do you need to carry out the comfort rounding?

Review Copy

- What supporting materials are needed?	
**3.3 Preliminary conclusion**	**3 min**
a. Preliminary conclusion of the interview. Complete? Adequate? Any additions?<br>b. What is important regarding this topic that has not yet been mentioned/asked, and would you like to tell us more about?	
**3.4 Closing and thanks**	**3 min**
a. How did you experience the interview? (feedback)<br>b. Interviewer explains the follow-up:<br>Reporting and member check (preferably) via email. Provides an opportunity to supplement or correct the typed report and ask for feedback for approval.<br>Implementation of comfort rounding and other interventions and follow-up research (interim measurements: T1, T2, and T3).<br>c. Thank colleagues and close.	

Review Copy

Focus groep interview protocol T1-T3*

Background

This interview protocol has been developed for the focus group interviews with nurses to determine whether comfort rounding with attention to mobility and food intake is feasible/applicable in practice and how nurses experience this.

Purpose of the interview

The purpose of the focus group interviews with nurses during T2 is:

- To gain insight into the hindering and facilitating factors for performing comfort rounding related to nutrition and movement.
- To gain insight into the experiences with performing comfort rounding (feasibility).
- To determine to what extent the intervention needs adjustment and to what extent support materials are needed to optimise comfort rounding.

Central themes

The focus group interview has 3 central themes:

● Facilitating and hindering factors regarding comfort rounding related to nutrition and movement.

● Feasibility of comfort rounding.

● Required support materials for optimizing the implementation of comfort rounding.

The themes are reflected in central questions. These questions address all aspects of the used implementation model (1). Where the discussion does not progress well or where certain topics are insufficiently covered, additional questions have been prepared.

**Semi structured focus group interviews**	
**3.1 Introduction + opening question**	**5-10 min**

Review Copy

Care for mobilisation and nutrition is essential for the recovery of surgical patients. Both individually and combined, mobility and adequate food intake can lead to, among other things, maintenance of function, improved self-care, shortened hospital stay, fewer complications, and have a positive impact on confidence in recovery and psychological well-being.<br>For this study, it is important to know how you, as a nurse, experience the care related to movement and nutrition since the implementation of comfort rounding.<br><br>**"Since the beginning of May 2022, comfort rounding has been implemented. Can you tell us how you experience performing the comfort rounding?"**	
**3.2 Interview sub topics**	**40-50 min**
**A.0 Feasibility of comfort rounding**<br>• **"Which aspects of the comfort rounding would you definitely like to keep? And why?"**<br>• **"And which aspects would you like to see changed? Why and how?"**<br>• **"What do you still need to be able to perform the comfort rounding?"**<br>• **"How does comfort rounding fit into the daily care you provide to patients? How does this process go?"**	
Additional questions	
- "How do you find the frequency of comfort rounding?"<br>- "How can we ensure that ALL nurses on the ward perform the comfort rounding?"	
**A.1. Attention**<br>You have just shared your experiences with performing comfort rounding in relation to mobility and nutrition (brief summary). Now, we would like to ask more specific questions about the use of comfort rounding.<br>Transition to attention and ask the question:	

Review Copy

• **"Have you noticed a change in attention to nutrition and mobility since the introduction of comfort rounding?" Ask for examples.**
**A.2. Encouraging/motivating**<br>You have just indicated how you pay attention to mobility and nutrition with your patients (brief summary). For some patients, mobilising and adequate food intake happens naturally, while for others it is less successful. This can have multiple causes, including a lack of patient motivation.<br>• **"In what ways does comfort rounding contribute to stimulating/motivating patients in mobilising and food intake? How do you notice this?"**
**A.3. Insight into mobility/nutrition behaviour (participation)**<br>Insight into mobility and nutrition behaviour gives you, as a nurse, an indication of how your patient's recovery is progressing.<br><br>• **"To what extent does comfort rounding contribute to gaining more insight into the movement and nutrition behaviour of patients?"**<br>• "How has this changed since the introduction of comfort rounding?"
**A.4. Patient participation**<br>We have discussed attention, patient motivation, and insight into the mobility and nutrition behaviour of your patients.<br><br>**"Do you find that comfort rounding helps in taking into account the preferences of your patients when making arrangements about movement and nutrition?"**
**A.4.a. Informing/education**<br>Transitioning from patient participation to:<br>**- "Are you sufficiently informed about performing comfort rounding? Examples."**<br>**- "Or do you lack information? If so, how come? What is needed?"**
**A.5. Knowledge/skills**

Review Copy

Your knowledge and skills are always with you when you talk to your patient, to inform but also to motivate/stimulate the patient.<br>• **"In what ways do your knowledge and skills contribute to the execution of the comfort rounding?"**
**B.1. Team attention**<br>We have just discussed very individually how you, as a nurse, pay attention to mobility and nutrition. In caring for your patient, you are part of a multidisciplinary team.<br>• **"Have you noticed any changes within the team regarding care and attention to nutrition, mobility, and patient participation since the implementation of comfort rounding? Why or why not?"**
Additional questions
- "What agreements have been made within the team regarding the content of the comfort rounding?"<br>- "What agreements have been made within the team regarding the practical execution of the comfort rounding?"<br>- "How does the team ensure continuity of care?"
**B.2. Role perception team**<br>Brief summary of team attention.<br>• **"Have any changes in collaboration with other disciplines been noticed since the introduction of comfort rounding?"**
Additional questions
- "Who do you think plays a role in performing comfort rounding? Should other people be involved as well?"<br>- "Is the care coordinated among the team? In what way?"

Review Copy

**B.3. Influence (role) family/relatives**<br>In caring for your patient, you also deal with concerns from family/relatives. "Do family/relatives influence the movement and nutrition behaviour of patients (positively/negatively)? How?"<br>• "Does the family influence the comfort rounding? Should they be involved?"	
**C.1. Organisation of care**<br>We have just discussed how attention is paid to movement and nutrition as a multidisciplinary team.<br>• **"In what ways does comfort rounding fit into the organisation of (multidisciplinary) care on the ward? What is going well and what could be improved?"**	
Additional questions	
- Is there sufficient time to perform comfort rounding?<br>- Do you feel supported by the rest of the team in performing the comfort rounding?	
**C.2. Environmental factors**<br>Research has shown that environmental factors also play an important role in movement and nutrition.<br>"How do you see this?"	
**3.3 Preliminary conclusion**	**3 min**
- Preliminary conclusion of the interview. Complete? Adequate? Any additions?<br>- What is important regarding this topic that has not yet been mentioned/asked, and would you like to tell us more about?	
**3.4 Thanks and close**	**3 min**
- How did you experience the interview? (feedback)<br>- Interviewer explains the follow-up:<br>Member check (preferably) via email.	

Review Copy

Explanation of interventions and follow-up research. Thank colleagues and close.

*T0 is the ‘baseline measurement’, T1 and T2 are ‘intermediate measurements’, and T3 is the ‘follow-up measurement’.

References:
1. Grol R, Bosch M, Wensing M. Development and selection of strategies for improving patient care. Improving Patient Care2013. p. 165-84.

Review Copy

Appendix D. Respondent characteristics of the focus group interviews and questionnaires

Table 1. Respondent characteristics of the focus group interviews

Study stage*	T0 n=8		T1 n=9		T2 n=8		T3 n=7	
**Hospital****	**H1**[#] n=4	**H2**[^] n=4	**H1**[#] n=5	**H2**[^] n=4	**H1**[#] n=3	**H2**[^] n=5	**H1**[#] n=3	**H2** n=4
**Age** (mean)	29,8	29,3	33,6	26,3	34,3	38	Missing	35,8
**Gender** (n)								
Male	0	0	1	0	0	0	0	1
Female	4	4	4	4	3	5	3	3
**Work experience as a nurse** (years)	8	5,5	Missing	4,5	15,3	14,8	15	13,3
**Highest educational level** (n)								
Vocational	1	1	4	0	0	2	1	1
Registered	2	3	0	3	1	3	0	3
Other	1	0	1	1	2	0	2	0

*T0 is the 'baseline measurement', T1 and T2 are 'intermediate measurements', and T3 is the 'follow-up measurement'

** H1=Hospital 1, H2=Hospital 2

[#] In Hospital 1, one respondent participated in focus group interviews at T0, T1, and T2; one respondent participated at T1 and T3; and one respondent participated at T2 and T3

^ In Hospital 2, one respondent participated in the focus group interviews T0 and T1, and one respondent participated at both T0 and T2.

Table 2. Respondent characteristics and results from questions 1-7 of the questionnaire

Question	Sample n=43 n (%)	Hospital 1 n=21 n (%)	Hospital 2 n=22 n (%)
**1. Gender**			
Male	4 (9%)	1 (5%)	3 (14%)
Female	38 (88%)	19 (91%)	19 (86%)
Unknown	1 (2%)	1 (5%)	0 (0%)
	n =43	n=21	n=22
**2. Age in years**			
≤20	2 (5%)	2 (10%)	0 (0%)
21–30	16 (37%)	6 (29%)	10 (46%)
31–40	10 (23%)	4 (19%)	6 (27%)
41–50	9 (21%)	4 (19%)	5 (23%)
51–60	3 (7%)	3 (14%)	0 (0%)

Review Copy

≥61	3 (7%)<br><br>n=43	2 (10%)<br><br>n=21	1 (5%)<br><br>n= 22
**3. Work experience as a nurse (in months)**<br>Median (IQR)*<br>Min, max	 <br><br><br>n=40	 84 (31,5–204)<br>12–432<br><br>n=19	 84 (48–216)<br>7–480<br><br>n=21
**4. Work experience on the ward (in months)**<br>Median (IQR)*<br>Min, max	 <br><br><br>n=39	 48 (12–144)<br>3–384<br><br>n=18	 60 (24–186)<br>3–480<br><br>n=21
**5. Contract hours (in hours)**<br>Median (IQR)*<br>Min, max	 <br><br><br>n=40	 32 (24–32)<br>12–36<br><br>n=18	 32 (24–32)<br>16–36<br><br>n=22
**6. Highest educational level**<br>Vocational nursing degree<br>Registered nursing degree<br>Other	 19 (44%)<br>23 (53%)<br>1 (2%)<br><br>n=43	 13 (62%)<br>8 (38%)<br>0 (0%)<br><br>n=21	 6 (27%)<br>15 (68%)<br>1 (5%)<br><br>n=22
**7. Conscious of comfort rounding?**<br>Yes<br>No	 38 (88%)<br>5 (11%)<br><br>n=43	 16 (76%)<br>5 (24%)<br><br>n=21	 22 (100%)<br>0 (0%)<br><br>n=22

*Median and interquartile range (IQR), measured according to Tukey's Hinges

Review Copy

Appendix A. Structure and content of comfort rounding

Schedule of comfort rounding at Hospital 1 in day shift and evening shift

Day shift		
**Time**	**Nutrition**	**Mobility**
7:15 – 9:00		• Discuss wishes and possibilities regarding ADL (Activities of Daily Living) and mobilization in the morning.<br>• Provide advice on alternatives/options.<br>• Discuss expectations and make agreements
8:30 – 9:00	• Patient is offered breakfast.	• Have the patient sit in the chair during breakfast and provide support if needed.
10.00 – 12.00	• Inquire about food intake compared to normal:<br>☐ More than usual<br>☐ Unchanged<br>☐ Less than usual<br><br>• Ask about:<br>☐ Appetite<br>☐ Taste<br>☐ Nausea/vomiting<br>☐ Swallowing difficulties<br>☐ Sore mouth<br>☐ Constipation/diarrhea<br>☐ Feeling full quickly<br><br>• If there are problems with food intake:<br>☐ Explain the (possible) cause<br>☐ Discuss wishes/possibilities<br>☐ Provide advice on alternatives/options<br>☐ Discuss expectations and make agreements (and follow through)	• Discuss with the patient how their mobility is and where support is needed:<br>☐ Use of aids<br>☐ Moving in bed<br>☐ Moving from lying on the back to sitting on the edge of the bed<br>☐ Moving from bed to chair<br>☐ Standing up from a chair<br>☐ Walking in the hospital room<br><br>• Provide support as needed based on previous agreements.
12:00 – 12:30	• Patient is offered lunch.	• Have the patient sit in the chair during lunch.
13.00 – 14.00	• Rest hour for patients.	• Rest hour for patients.
14.00 – 15.00	• Check the food list (if agreed upon).<br>• Evaluate expectations and agreements made, and adjust them if necessary, in consultation with the patient.<br>• Report in SBARR/ADL.	• Evaluate expectations and agreements made, and adjust them if necessary, in consultation with the patient.<br>• Provide support as needed based on agreements made.<br>• Report in SBARR/ADL.
15.00 – 15.45	• Discuss food/fluid intake and expectations/agreements made during bedside handover.	• Discuss mobility and expectations/agreements made during bedside handover.

Review Copy

**Evening shift**		
15.00 – 15.45	• Discuss food/fluid intake and expectations/agreements made during bedside handover.	• Discuss mobility and expectations/agreements made during bedside handover.
17.00 – 18.00	• Patient is offered dinner.	• Have the patient sit in the chair during dinner and provide support if needed.
18.00 – 22.00	• Check the food list (if agreed upon).<br>• Evaluate with the patient how eating and drinking went today:<br>□ More than usual<br>□ Unchanged<br>□ Less than usual<br>• Ask about:<br>□ Appetite<br>□ Taste<br>□ Nausea/vomiting<br>□ Swallowing difficulties<br>□ Sore mouth<br>□ Constipation/diarrhea<br>□ Feeling full quickly<br>• If there are problems with food intake:<br>□ Explain the (possible) cause<br>□ Discuss wishes/possibilities<br>□ Provide advice on alternatives/options<br>• Report in SBARR/ADL.	• Provide support as needed based on agreements made.<br>• Evaluate with the patient how their mobility has been today:<br>□ Use of aids<br>□ Moving in bed<br>□ Moving from lying on the back to sitting on the edge of the bed<br>□ Moving from bed to chair<br>□ Standing up from a chair<br>□ Walking in the hospital room<br>• Report in SBARR/ADL.

<u>Schedule of comfort rounding at Hospital 2 in day shift and evening shift</u>

Time	Nutrition	Mobility
8.00	• Inform the patient how many calories and proteins they consumed yesterday, when closing the fluid balance and food registration.<br>• Inform the patient how many calories and proteins they need for recovery, and what they should ideally aim for during their stay.	• Discuss with the patient their wishes and possibilities regarding mobilisation in the morning (including ADL - Activities of Daily Living).
9.00 – 11.30	• Discuss mutual expectations regarding food intake with the patient.<br>• Check if the patient is familiar with the FoodforCare concept; if not, ask the nutrition assistants to explain it.<br>• Consult with the patient on their food intake goals for the day and provide nursing advice accordingly.<br>• Inform the patient about proteins (awareness: why they are needed, where proteins are found).<br>• Coordinate with the patient if and when they need help making choices about food or with eating and drinking themselves<br><br>*<u>If patients do not eat and drink sufficiently:</u>*<br>• Discuss with the patient the reasons why they are not eating and drinking enough.<br>• Inform and discuss alternatives or possible solutions with the patient (different food products, (other) nutritional drinks, anti-emetics, food from home).	• Carry out what was agreed upon with the patient in the morning (e.g., washing at the edge of the bed, showering, sitting in the chair)

Review Copy

12.00 – 15.00	• Provide support during meals if necessary (consider the position and place where the patient eats).<br>• Evaluate with the patient how eating and drinking went during breakfast and lunch.<br>• Inform the patient about how many calories and proteins they have consumed so far.<br>• Adjust previously made expectations in consultation with the patient, if necessary	• Check if the agreements made this morning regarding mobilisation are still valid/feasible.<br>• Discuss with the patient their wishes and possibilities regarding mobilisation in the afternoon (consider 'eating at the table' and mobilising with family, for example).<br>• Carry out what was agreed upon with the patient in the afternoon.
17.00	• Provide support during meals if necessary (consider the position and place where the patient eats).	• Discuss with the patient their wishes and possibilities regarding mobilization in the evening (include ADL such as brushing teeth, for example), and carry this out
20.00 – 22.00	• Evaluate with the patient how eating and drinking went today.<br>• Inform the patient about how many calories and proteins they have consumed today	• Carry out what was agreed upon with the patient at 17:00.<br>• Evaluate with the patient how their mobilization went today.

Review Copy

Appendix C. Questionnaire on comfort rounding during the follow-up measurement (T3)

Part 1: General questions

For multiple-choice questions, please select the answer that best fits you.

1. **I identify myself as**:

☐ Male
☐ Female
☐ Other, namely_______________________________
☐ Prefer not to say

2. **What is your age?**

☐ ≤20 years
☐ 21-30 years
☐ 31-40 years
☐ 41-50 years
☐ 51-60 years
☐ ≥61 years
☐ Prefer not to say

3. **How long have you been working as a nurse?**
Calculate from the moment you obtained your first nursing diploma. Estimate if you do not know the exact number.

☐ ______________ years If <1 years: ______________ months
☐ Prefer not to say

4. **How long have you been working on nursing ward X at Hospital 1 or Hospital 2?**
Estimate if you do not know the exact number.

☐ ______________ years If <1 years: ______________ months
☐ Prefer not to say

5. **How many contract hours do you have on this nursing ward?**

☐ ______________ hours
☐ Prefer not to say

6. **What is your highest obtained nursing education level?**

☐ MBO-V (vocational nursing degree)
☐ Inservice-education (vocational nursing degree)
☐ HBO-V (bachelor's nursing degree)
☐ Other, namely ______________

Review Copy

Part 2: Execution of comfort rounding

In this part of the questionnaire, you indicate how you perform the comfort rounding. For multiple-choice questions, please select the answer that best fits you.

For your information: Comfort rounding = "structured attention to nutrition, movement, and patient participation by nurses with and for patients on the ward."

7. I am aware that comfort rounding for nutrition and mobility is being conducted on my ward.

☐ Yes (continue to question 8 on page 3)

☐ No (continue to question 12 on page 5)

8. Indicate to what extent you agree with the following statements by placing a cross in the column with the answer that best matches your opinion. You can provide an explanation for your answer for each statement.

	Strongly disagree	Disagree	Neutral	Agree	Strongly agree	Explanation
1. Comfort rounding helps me to have structured attention to nutrition and mobility.						
2. I pay attention to nutrition and mobility in consultation with the patient.						
3. The way comfort rounding is implemented fits well with practice.						
4. Comfort rounding has no added value for me.						
5. I have sufficient time to perform comfort rounding.						

Review Copy

6. I use the patient record (EPIC/SAP) when performing comfort rounding.						
7. Performing comfort rounding is not one of my priorities during my shift.						

Review Copy

9. Think about the day shifts of the past four weeks. How often do you perform comfort rounding during the day shifts?

- ☐ Always
- ☐ Usually
- ☐ About half of the time
- ☐ Rarely
- ☐ Never

10. Think about the evening shifts of the past four weeks. How often do you perform comfort rounding during the evening shifts?

- ☐ Always
- ☐ Usually
- ☐ About half of the time
- ☐ Rarely
- ☐ Never

Review Copy

11. Indicate to what extent you pay attention to the following aspects <u>during comfort rounding</u> by placing a cross in the column with the answer that best matches your opinion. You can provide an explanation for your answer for each statement.

	Never	Rarely	Seldom	Often	Very often/ always	Explanation
1. I discuss the nutritional and mobility status with the patient *(e.g., creating moments to ask how eating and moving are going, and about complaints)*						
2. I inform the patient about the importance of nutrition and mobility *(e.g., providing oral information; sharing information sources)*						
3. I support the patient in performing nutritional and mobility interventions *(e.g., offering physical support/aids; making the environment suitable)*						
4. I stimulate and/or motivate the patient to eat and move *(e.g., expressing expectations to the patient, giving encouragement)*						
5. I give advice and discuss possibilities/alternatives regarding nutrition and mobility with the patient *(e.g., discussing questions; advising specific food or drink)*						
6. I discuss wishes and expectations and/or gauge needs regarding nutrition						

	Never	Rarely	Seldom	Often	Very often/ always	Explanation
and mobility with the patient *(e.g., discussing what the patient can and wants to do)*						
7. I make agreements with the patient about food intake and movement						
8. I carry out the agreements I have made with the patient						
9. I evaluate the care regarding nutrition and mobility with the patient *(e.g., how the care/execution of agreements went)*						

Review Copy

Note: only fill out questions 12 and 13 if you answered 'no' to question 7.

12. **You indicated in question 7 that you are not aware of the existence of comfort roundings on your ward. Explain how and/or where this information provision can be improved.**

____

____

____

With comfort rounding, nurses regularly visit patients to pay attention to nutrition, mobility, and patient participation. Comfort rounding takes place at fixed times and/or frequencies, with structured attention to essential care aspects as a result. Through comfort rounding, care regarding nutrition, mobility, and patient participation may be improved.

13. **Does the above concept of "comfort rounding" appeal to you to apply in the daily care of patients? Circle 'yes' or 'no' and explain your answer.**

Yes / No , because ________________________________

____

____

End of questionnaire

Do you have any questions or comments? You can note them below:

____

____

____

Thank you for completing this questionnaire!

Review Copy

COREQ (COnsolidated criteria for REporting Qualitative research) Checklist

A checklist of items that should be included in reports of qualitative research. You must report the page number in your manuscript where you consider each of the items listed in this checklist. If you have not included this information, either revise your manuscript accordingly before submitting or note N/A.

Topic	Item No.	Guide Questions/Description	Reported on Page No.
**Domain 1: Research team and reflexivity**			
Personal characteristics			
Interviewer/facilitator	1	Which author/s conducted the interview or focus group?	10
Credentials	2	What were the researcher's credentials? E.g. PhD, MD	10
Occupation	3	What was their occupation at the time of the study?	10
Gender	4	Was the researcher male or female?	10
Experience and training	5	What experience or training did the researcher have?	10
Relationship with participants			
Relationship established	6	Was a relationship established prior to study commencement?	n/a
Participant knowledge of the interviewer	7	What did the participants know about the researcher? e.g. personal goals, reasons for doing the research	n/a
Interviewer characteristics	8	What characteristics were reported about the inter viewer/facilitator? e.g. Bias, assumptions, reasons and interests in the research topic	23
**Domain 2: Study design**			
Theoretical framework			
Methodological orientation and Theory	9	What methodological orientation was stated to underpin the study? e.g. grounded theory, discourse analysis, ethnography, phenomenology, content analysis	6
Participant selection			
Sampling	10	How were participants selected? e.g. purposive, convenience, consecutive, snowball	8
Method of approach	11	How were participants approached? e.g. face-to-face, telephone, mail, email	8
Sample size	12	How many participants were in the study?	13
Non-participation	13	How many people refused to participate or dropped out? Reasons?	n/a
Setting			
Setting of data collection	14	Where was the data collected? e.g. home, clinic, workplace	7
Presence of non-participants	15	Was anyone else present besides the participants and researchers?	9,10
Description of sample	16	What are the important characteristics of the sample? e.g. demographic data, date	Appendix D
Data collection			
Interview guide	17	Were questions, prompts, guides provided by the authors? Was it pilot tested?	10
Repeat interviews	18	Were repeat inter views carried out? If yes, how many?	n/a
Audio/visual recording	19	Did the research use audio or visual recording to collect the data?	10
Field notes	20	Were field notes made during and/or after the inter view or focus group?	10
Duration	21	What was the duration of the inter views or focus group?	13
Data saturation	22	Was data saturation discussed?	11
Transcripts returned	23	Were transcripts returned to participants for comment and/or	10

Review Copy

Topic	Item No.	Guide Questions/Description	Reported on Page No.
		correction?	
**Domain 3: analysis and findings**			
Data analysis			
Number of data coders	24	How many data coders coded the data?	12
Description of the coding tree	25	Did authors provide a description of the coding tree?	11-12
Derivation of themes	26	Were themes identified in advance or derived from the data?	11-12
Software	27	What software, if applicable, was used to manage the data?	12
Participant checking	28	Did participants provide feedback on the findings?	10
Reporting			
Quotations presented	29	Were participant quotations presented to illustrate the themes/findings? Was each quotation identified? e.g. participant number	13-19
Data and findings consistent	30	Was there consistency between the data presented and the findings?	20
Clarity of major themes	31	Were major themes clearly presented in the findings?	13-20
Clarity of minor themes	32	Is there a description of diverse cases or discussion of minor themes?	13-20

Developed from: Tong A, Sainsbury P, Craig J. Consolidated criteria for reporting qualitative research (COREQ): a 32-item checklist for interviews and focus groups. *International Journal for Quality in Health Care*. 2007. Volume 19, Number 6: pp. 349 – 357

Once you have completed this checklist, please save a copy and upload it as part of your submission. DO NOT include this checklist as part of the main manuscript document. It must be uploaded as a separate file.

Review Copy

Supplement 3: Checklist for Reporting Of Survey Studies (CROSS)

Section/topic	Item	Item description	Reported on page #
**Title and abstract**			
Title and abstract	1a	State the word “survey” along with a commonly used term in title or abstract to introduce the study’s design.	
	1b	Provide an informative summary in the abstract, covering background, objectives, methods, findings/results, interpretation/discussion, and conclusions.	**1**
**Introduction**			
Background	2	Provide a background about the rationale of study, what has been previously done, and why this survey is needed.	**4-6**
Purpose/aim	3	Identify specific purposes, aims, goals, or objectives of the study.	**6**
**Methods**			
Study design	4	Specify the study design in the methods section with a commonly used term (e.g., cross-sectional or longitudinal).	**6-7**
Data collection methods	5a	Describe the questionnaire (e.g., number of sections, number of questions, **10** number and names of instruments used).	
	5b	Describe all questionnaire instruments that were used in the survey to measure particular concepts. Report target population, reported validity and reliability information, scoring/classification procedure, and reference links (if any). **n/a**	
	5c	Provide information on pretesting of the questionnaire, if performed (in the article or in an online supplement). Report the method of pretesting, number of times questionnaire was pre-tested, number and demographics of participants used for pretesting, and the level of similarity of demographics between pre-testing participants and sample population. **n/a**	
	5d	Questionnaire if possible, should be fully provided (in the article, or as appendices or as an online supplement). **Appendix C**	
Sample characteristics	6a	Describe the study population (i.e., background, locations, eligibility criteria for participant inclusion in survey, exclusion criteria). **8**	
	6b	Describe the sampling techniques used (e.g., single stage or multistage sampling, simple random sampling, stratified sampling, cluster sampling, convenience sampling). Specify the locations of sample participants whenever clustered sampling was applied. **8**	
	6c	Provide information on sample size, along with details of sample size calculation. **Appendix D**	**11+**
	6d	Describe how representative the sample is of the study population (or target population if possible), particularly for population-based surveys.	**n/a**

Review Copy

Survey administration	7a	Provide information on modes of questionnaire administration, including the type and number of contacts, the location where the survey was conducted (e.g., outpatient room or by use of online tools, such as SurveyMonkey).<br>**10-11**
	7b	Provide information of survey's time frame, such as periods of recruitment, exposure, and follow-up days.<br>**11**
	7c	Provide information on the entry process:<br>**n/a**<br>–>For non-web-based surveys, provide approaches to minimize human error in data entry.<br>–>For web-based surveys, provide approaches to prevent "multiple participation" of participants.
Study preparation	8	Describe any preparation process before conducting the survey (e.g., interviewers' training process, advertising the survey).<br>**n/a**
Ethical considerations	9a	Provide information on ethical approval for the survey if obtained, including informed consent, institutional review board [IRB] approval, Helsinki declaration, and good clinical practice [GCP] declaration (as appropriate).<br>**12**
	9b	Provide information about survey anonymity and confidentiality and describe what mechanisms were used to protect unauthorized access.<br>**10**
Statistical analysis	10a	Describe statistical methods and analytical approach. Report the statistical software that was used for data analysis.<br>**11**
	10b	Report any modification of variables used in the analysis, along with reference (if available).<br>**n/a**
	10c	Report details about how missing data was handled. Include rate of missing items, missing data mechanism (i.e., missing completely at random [MCAR], missing at random [MAR] or missing not at random [MNAR]) and methods used to deal with missing data (e.g., multiple imputation).<br>**n/a**
	10d	State how non-response error was addressed.<br>**n/a**
	10e	For longitudinal surveys, state how loss to follow-up was addressed. **n/a**
	10f	Indicate whether any methods such as weighting of items or propensity scores have been used to adjust for non-representativeness of the sample.<br>**n/a**
	10g	Describe any sensitivity analysis conducted.<br>**n/a**
**Results**		

Review Copy

Respondent characteristics	11a	Report numbers of individuals at each stage of the study. Consider using a flow diagram, if possible. **Appendix D**
	11b	Provide reasons for non-participation at each stage, if possible. **n/a**
	11c	Report response rate, present the definition of response rate or the formula used to calculate response rate. **n/a**
	11d	Provide information to define how unique visitors are determined. Report number of unique visitors along with relevant proportions (e.g., view proportion, participation proportion, completion proportion). **Appendix D**
Descriptive results	12	Provide characteristics of study participants, as well as information on potential confounders and assessed outcomes. **Appendix D, 13, 23**
Main findings	13a	Give unadjusted estimates and, if applicable, confounder-adjusted estimates along with 95% confidence intervals and p-values. **n/a**
	13b	For multivariable analysis, provide information on the model building process, model fit statistics, and model assumptions (as appropriate). **n/a**
	13c	Provide details about any sensitivity analysis performed. If there are considerable amount of missing data, report sensitivity analyses comparing the results of complete cases with that of the imputed dataset (if possible). **n/a**
**Discussion**		
Limitations	14	Discuss the limitations of the study, considering sources of potential biases and imprecisions, such as non-representativeness of sample, study design, important uncontrolled confounders. **22,23**
Interpretations	15	Give a cautious overall interpretation of results, based on potential biases and imprecisions and suggest areas for future research. **20, 21**
Generalizability	16	Discuss the external validity of the results. **22,23**
**Other sections**		
Role of funding source	17	State whether any funding organization has had any roles in the survey's design, implementation, and analysis. **Title page**
Conflict of interest	18	Declare any potential conflict of interest. **Title page**
Acknowledgements	19	Provide names of organizations/persons that are acknowledged along with their contribution to the research. **Title page**

Review Copy

Review Copy

Implications for the profession and/or patient care:

Comfort roundings' concept does not align well with current nursing practice. Greater tailoring to nurses' preferences or alternative approaches to structuring personalised nutritional and mobility care are recommended.

Review Copy
