# Supplementary material for: Evaluating Nurses' Perspectives on the Acceptability and Practicality of Comfort Rounding for Personalised Nutritional and Mobility Care in Surgical Wards: A Mixed‐Methods Feasibility Study
Source: J Adv Nurs. 2025 Dec 21;82(8):8158–71. doi: 10.1111/jan.70462 (PMC13356403; doi:10.1111/jan.70462)
Supplement: Supplementary file 3 — Appendix C. [file JAN-82-8158-s005.docx]

**Appendix C. Questionnaire on comfort rounding during the follow-up measurement (T3)**

**Part 1: General questions**

For multiple-choice questions, please select the answer that best fits you.

1. **I identify myself as**:

- Male
- Female
- Other, namely____________________________
- Prefer not to say

1. **What is your age?**

- ≤20 years
- 21-30 years
- 31-40 years
- 41-50 years
- 51-60 years
- ≥61 years
- Prefer not to say

1. **How long have you been working as a nurse?**

*Calculate from the moment you obtained your first nursing diploma. Estimate if you do not know the exact number.*

- _____________ years If <1 years: _____________ months
- Prefer not to say

1. **How long have you been working on nursing ward X at Hospital 1 or Hospital 2?**

*Estimate if you do not know the exact number.*

- _____________ years If <1 years: _____________ months
- Prefer not to say

1. **How many contract hours do you have on this nursing ward?**

- _____________ hours
- Prefer not to say

1. **What is your highest obtained nursing education level?**

- MBO-V (vocational nursing degree)
- Inservice-education (vocational nursing degree)
- HBO-V (bachelor’s nursing degree)
- Other, namely _____________

**Part 2: Execution of comfort rounding**

In this part of the questionnaire, you indicate how you perform the comfort rounding. For multiple-choice questions, please select the answer that best fits you.

For your information: Comfort rounding = “structured attention to nutrition, movement, and patient participation by nurses with and for patients on the ward.”

1. **I am aware that comfort rounding for nutrition and mobility is being conducted on my ward.**

- Yes (continue to question 8 on page 3)
- No (continue to question 12 on page 5)

1. **Indicate to what extent you agree with the following statements by placing a cross in the column with the answer that best matches your opinion. You can provide an explanation for your answer for each statement.**

|  | Strongly disagree | Disagree | Neutral | Agree | Strongly agree | Explanation |
| --- | --- | --- | --- | --- | --- | --- |
| 1. Comfort rounding helps me to have structured attention to nutrition and mobility. |  |  |  |  |  |  |
| 2. I pay attention to nutrition and mobility in consultation with the patient. |  |  |  |  |  |  |
| 3. The way comfort rounding is implemented fits well with practice. |  |  |  |  |  |  |
| 4. Comfort rounding has no added value for me. |  |  |  |  |  |  |
| 5. I have sufficient time to perform comfort rounding. |  |  |  |  |  |  |
| 6. I use the patient record (EPIC/SAP) when performing comfort rounding. |  |  |  |  |  |  |
| 7. Performing comfort rounding is not one of my priorities during my shift. |  |  |  |  |  |  |

1. **Think about the day shifts of the past four weeks. How often do you perform comfort rounding during the day shifts?**

- Always
- Usually
- About half of the time
- Rarely
- Never

1. **Think about the evening shifts of the past four weeks. How often do you perform comfort rounding during the evening shifts?**

- Always
- Usually
- About half of the time
- Rarely
- Never

1. **Indicate to what extent you pay attention to the following aspects during comfort rounding by placing a cross in the column with the answer that best matches your opinion. You can provide an explanation for your answer for each statement.**

|  | Never | Rarely | Seldom | Often | Very often/  always | Explanation |
| --- | --- | --- | --- | --- | --- | --- |
| 1. I discuss the nutritional and mobility status with the patient *(e.g., creating moments to ask how eating and moving are going, and about complaints)* |  |  |  |  |  |  |
| 2. I inform the patient about the importance of nutrition and mobility *(e.g., providing oral information; sharing information sources)* |  |  |  |  |  |  |
| 3. I support the patient in performing nutritional and mobility interventions *(e.g., offering physical support/aids; making the environment suitable)* |  |  |  |  |  |  |
| 4. I stimulate and/or motivate the patient to eat and move *(e.g., expressing expectations to the patient, giving encouragement)* |  |  |  |  |  |  |
| 5. I give advice and discuss possibilities/alternatives regarding nutrition and mobility with the patient *(e.g., discussing questions; advising specific food or drink)* |  |  |  |  |  |  |
| 6. I discuss wishes and expectations and/or gauge needs regarding nutrition and mobility with the patient *(e.g., discussing what the patient can and wants to do)* |  |  |  |  |  |  |
| 7. I make agreements with the patient about food intake and movement |  |  |  |  |  |  |
| 8. I carry out the agreements I have made with the patient |  |  |  |  |  |  |
| 9. I evaluate the care regarding nutrition and mobility with the patient *(e.g., how the care/execution of agreements went)* |  |  |  |  |  |  |

Note: only fill out questions 12 and 13 if you answered 'no' to question 7.

1. **You indicated in question 7 that you are not aware of the existence of comfort roundings on your ward. Explain how and/or where this information provision can be improved.**

|  |
| --- |
|  |
|  |

With comfort rounding, nurses regularly visit patients to pay attention to nutrition, mobility, and patient participation. Comfort rounding takes place at fixed times and/or frequencies, with structured attention to essential care aspects as a result. Through comfort rounding, care regarding nutrition, mobility, and patient participation may be improved.

1. **Does the above concept of "comfort rounding" appeal to you to apply in the daily care of patients? Circle 'yes' or 'no' and explain your answer.**

| Yes / No , because |
| --- |
|  |
|  |

**End of questionnaire**

Do you have any questions or comments? You can note them below:

|  |
| --- |
|  |
|  |

**Thank you for completing this questionnaire!**
