# Supplementary material for: Evaluating Nurses' Perspectives on the Acceptability and Practicality of Comfort Rounding for Personalised Nutritional and Mobility Care in Surgical Wards: A Mixed‐Methods Feasibility Study
Source: J Adv Nurs. 2025 Dec 21;82(8):8158–71. doi: 10.1111/jan.70462 (PMC13356403; doi:10.1111/jan.70462)
Supplement: Supplementary file 4 — Appendix D. [file JAN-82-8158-s002.docx]

**Appendix D. Respondent characteristics of the focus group interviews and questionnaires**

**Table 1. Respondent characteristics of the focus group interviews**

| **Study stage*** | **T0**  n=8 | | **T1**  n=9 | | **T2**  n=8 | | **T3**  n=7 | |
| --- | --- | --- | --- | --- | --- | --- | --- | --- |
| **Hospital**** | **H1^#^**  n=4 | **H2^^^**  n=4 | **H1^#^**  n=5 | **H2^^^**  n=4 | **H1^#^**  n=3 | **H2^^^**  n=5 | **H1^#^**  n=3 | **H2**  n=4 |
| **Age** (mean) | 29,8 | 29,3 | 33,6 | 26,3 | 34,3 | 38 | Missing | 35,8 |
| **Gender** (n)  Male  Female | 0  4 | 0  4 | 1  4 | 0  4 | 0  3 | 0  5 | 0  3 | 1  3 |
| **Work experience as a nurse** (years) | 8 | 5,5 | Missing | 4,5 | 15,3 | 14,8 | 15 | 13,3 |
| **Highest educational level (**n)  Vocational  Registered  Other | 1  2  1 | 1 3  0 | 4  0  1 | 0  3  1 | 0  1  2 | 2  3  0 | 1  0  2 | 1  3  0 |

*T0 is the ‘baseline measurement’, T1 and T2 are ‘intermediate measurements’, and T3 is the ‘follow-up measurement’
** H1=Hospital 1, H2=Hospital 2
^#^ In Hospital 1, one respondent participated in focus group interviews at T0, T1, and T2; one respondent participated at T1 and T3; and one respondent participated at T2 and T3
^ In Hospital 2, one respondent participated in the focus group interviews T0 and T1, and one respondent participated at both T0 and T2.

**Table 2. Respondent characteristics and results from questions 1-7 of the questionnaire**

| **Question** | **Sample**  n=43  n (%) | **Hospital 1**  n=21  n (%) | **Hospital 2**  n=22  n (%) |
| --- | --- | --- | --- |
| 1. **Gender**   Male  Female  Unknown | 4 (9%)  38 (88%)  1 (2%)  n =43 | 1 (5%)  19 (91%)  1 (5%)  n=21 | 3 (14%)  19 (86%)  0 (0%)  n=22 |
| 1. **Age in years**   ≤20  21─30  31─40  41─50  51─60  ≥61 | 2 (5%)  16 (37%)  10 (23%)  9 (21%)  3 (7%)  3 (7%)  n=43 | 2 (10%)  6 (29%)  4 (19%)  4 (19%)  3 (14%)  2 (10%)  n=21 | 0 (0%)  10 (46%)  6 (27%)  5 (23%)  0 (0%)  1 (5%)  n= 22 |
| 1. **Work experience as a nurse (in months)**   Median (IQR)*  Min, max | n=40 | 84 (31,5─204)  12─432  n=19 | 84 (48─216)  7─480  n=21 |
| 1. **Work experience on the ward (in months)**   Median (IQR)*  Min, max | n=39 | 48 (12─144)  3─384  n=18 | 60 (24─186)  3─480  n=21 |
| 1. **Contract hours (in hours)**   Median (IQR)*  Min, max | n=40 | 32 (24─32)  12─36  n=18 | 32 (24─32)  16─36  n=22 |
| 1. **Highest educational level**   Vocational nursing degree  Registered nursing degree  Other | 19 (44%)  23 (53%)  1 (2%)  n=43 | 13 (62%)  8 (38%)  0 (0%)  n=21 | 6 (27%)  15 (68%)  1 (5%)  n=22 |
| 1. **Conscious of comfort rounding?**   Yes  No | 38 (88%)  5 (11%)  n=43 | 16 (76%)  5 (24%)  n=21 | 22 (100%)  0 (0%)  n=22 |

*Median and interquartile range (IQR), measured according to Tukey’s Hinges
